# Nucleoporin 153 deficiency in adult neural stem cells defines a pathological protein-network signature and defective neurogenesis in a mouse model of AD

**DOI:** 10.1186/s13287-024-03805-1

**Published:** 2024-09-03

**Authors:** Claudia Colussi, Alessia Bertozzi, Lucia Leone, Marco Rinaudo, Raimondo Sollazzo, Federica Conte, Elena Paccosi, Luca Nardella, Giuseppe Aceto, Domenica Donatella Li Puma, Cristian Ripoli, Maria Gabriella Vita, Camillo Marra, Marcello D’Ascenzo, Claudio Grassi

**Affiliations:** 1 Istituto di Analisi dei Sistemi ed Informatica “Antonio Ruberti” (IASI) – CNR , National Research Council, Via dei Taurini 19, Rome, 00185 Italy; 2 Department of Neuroscience, Università Cattolica del Sacro Cuore, Rome, 00168 Italy; 3 Fondazione Policlinico Universitario Agostino Gemelli IRCCS, Rome, 00168 Italy

**Keywords:** Nucleoporin, Neurogenesis, Alzheimer’s disease, Organoids

## Abstract

**Background:**

Reduction of adult hippocampal neurogenesis is an early critical event in Alzheimer’s disease (AD), contributing to progressive memory loss and cognitive decline. Reduced levels of the nucleoporin 153 (Nup153), a key epigenetic regulator of NSC stemness, characterize the neural stem cells isolated from a mouse model of AD (3×Tg) (AD-NSCs) and determine their altered plasticity and gene expression.

**Methods:**

Nup153-regulated mechanisms contributing to NSC function were investigated: (1) in cultured NSCs isolated from AD and wild type (WT) mice by proteomics; (2) in vivo by lentiviral-mediated delivery of Nup153 or GFP in the hippocampus of AD and control mice analyzing neurogenesis and cognitive function; (3) in human iPSC-derived brain organoids obtained from AD patients and control subjects as a model of neurodevelopment.

**Results:**

Proteomic approach identified Nup153 interactors in WT- and AD-NSCs potentially implicated in neurogenesis regulation. Gene ontology (GO) analysis showed that Nup153-bound proteins in WT-NSCs were involved in RNA metabolism, nuclear import and epigenetic mechanisms. Nup153-bound proteins in AD-NSCs were involved in pathways of neurodegeneration, mitochondrial dysfunction, proteasomal processing and RNA degradation. Furthermore, recovery of Nup153 levels in AD-NSCs reduced the levels of oxidative stress markers and recovered proteasomal activity. Lentiviral-mediated delivery of Nup153 in the hippocampal niche of AD mice increased the proliferation of early progenitors, marked by BrdU/DCX and BrdU/PSANCAM positivity and, later, the integration of differentiating neurons in the cell granule layer (BrdU/NeuN^+^ cells) compared with GFP-injected AD mice. Consistently, Nup153-injected AD mice showed an improvement of cognitive performance in comparison to AD-GFP mice at 1 month after virus delivery assessed by Morris Water Maze. To validate the role of Nup153 in neurogenesis we took advantage of brain organoids derived from AD-iPSCs characterized by fewer neuroepithelial progenitor loops and reduced differentiation areas. The upregulation of Nup153 in AD organoids recovered the formation of neural-like tubes and differentiation.

**Conclusions:**

Our data suggest that the positive effect of Nup153 on neurogenesis is based on a complex regulatory network orchestrated by Nup153 and that this protein is a valuable disease target.

**Supplementary Information:**

The online version contains supplementary material available at 10.1186/s13287-024-03805-1.

## Introduction

Adult neurogenesis is a process occurring in specific niches of the postnatal brain where specialized neural stem cells (NSCs), activated by physiological stimuli or injury, give rise to new neurons and glial cells that will integrate within the neural circuits, thereby supporting hippocampal plasticity, memory and learning processes [1–4]. Although recent research has started to unveil the contribution of different regions of the adult brain [5–7] to neurogenesis, the most studied neurogenic niches are the subventricular zone (SVZ) of the lateral ventricles and the subgranular zone (SGZ) of the dentate gyrus (DG) of the hippocampus for the particular abundance of the phenomenon occurring in these brain areas as well as for their functional impact on learning and memory that is markedly impoverished in several neurological disorders including Alzheimer’s disease (AD). AD is a neurodegenerative disease characterized by accumulation of misfolded and toxic proteins, amyloid-β (Aβ) and tau, and progressive memory loss [8]. An increasing body of research has revealed that cognitive deficits in AD correlate with an impairment of adult hippocampal neurogenesis [9]. For example, reduction of early progenitors and young differentiating neurons (marked as DCX/PSANCAM, DCX/Prox1, and DCX/βIII-tubulin double-positive cells) has been observed in AD patients [10, 11]. Further, many studies using different AD animal models have confirmed the contribution of reduced neurogenesis to early AD dysfunction [12–14] suggesting that targeting NSC function could be a way to boost endogenous brain repair. Nevertheless, the effectiveness of this approach is hampered by the poor understanding of mechanisms that control the function of these cells.

Among these mechanisms, epigenetic regulation has a major role in NSC function tuning stemness/multipotency, fate specification and differentiation into mature cells. Epigenetic mechanisms are dynamic, reversible and heritable changes of chromatin that do not involve DNA sequence and include changes in histone post-translational modifications, non-coding RNA and DNA methylation that modify the transcription of genes [15]. Furthermore, epigenetic machinery also controls chromatin topological changes thus allowing the formation and maintenance of long-range chromatin loops that are crucial for expression of genes implicated in development and differentiation [16]. Lately, nucleoporins (Nups), which are components of nuclear pore channels and regulators of nucleus-cytoplasmic transport, have emerged as novel partners/coordinators of these epigenetic mechanisms and they have been identified as crucial regulators of embryonic and adult NSC function [17].

There are about 30 different Nups that form cylindrical channels embedded in the nuclear envelope. These pores are selectively permeable barriers that regulate the transport of proteins and RNAs (e.g. mRNA, microRNA). In addition, Nups act as gathering platform for chromatin and its co-regulators and/or directly bind to specific gene regions modulating recruitment and chromatin positioning of transcription factors and chromatin-modifying enzymes such as histone deacetylases (HDACs) or histone acetyl transferases (HATs) important for histone modifications and chromatin conformation [18–20].

Although Nup’s role in stem cell regulation has been mostly studied in embryonic stem cells (ESC), several works are unveiling Nup’s critical significance in adult NSCs. For example, Nup133 binds to chromatin remodeling enzymes to turn on genes involved in neural induction [21] and Nup98 is a gene activator dynamically interacting with lineage-specific genes in ESCs induced towards neural differentiation. Other studies have enlightened the master role of Nup153 in ESCs and in NSCs through the regulation of hundreds of genes implicated in stemness maintenance or fate determination [22, 23] or through the regulation of nuclear architecture [24]. According to the chromatin region that Nup153 binds it functions as an activator or silencer of transcription in coordination with the transcription factor Sox2 [22]. We further extended the role of Nup153 in physiopathology since we discovered that hippocampal NSCs isolated from an animal model of AD, the 3×Tg mice (AD-NSCs), show lower levels of Nup153 protein and that this deficiency affects Sox2 function and neurogenesis in vitro [25]. AD-NSCs indeed showed impaired proliferation, differentiation and migration that were recovered by the overexpression of Nup153. Furthermore, AD-NSCs expressing Nup153 differentiated into more mature neurons than their control AD-NSCs. These findings have prompted us to explore in depth the molecular pathways that Nup153 regulates in adult NSCs and to validate its role in neurogenesis by in vivo experiments and assessment of development of brain organoids derived from AD and control iPSCs.

## Methods

### Neural stem cell isolation

NSCs were prepared and cultured according to previously published protocols. Briefly, newborn (0–1 day old) WT and 3×Tg mice were anesthetized by deep hypothermia followed by decapitation to obtain the brain and microdissect the hippocampal regions. Tissues were finely minced and digested by accutase (in DPBS, 0.5 mM EDTA; Innovative Cell Tecnologies, Inc., San Diego, CA, USA) at 37 °C for 30 min. After centrifugation, cells were dissociated and resuspended in NeurobasalA medium, supplemented with 2% B27 without vitamin A (Gibco, Grand Island, NY, USA), Glutamax (0.5 mM; Invitrogen, Carlsbad, CA), mouse fibroblast growth factor 2 (FGF2, 10 ng/mL; Invitrogen), epidermal growth factor (EGF, 10 ng/mL; Invitrogen) and mouse platelet-derived growth factor bb (PDGFbb, 10 ng/mL; Invitrogen). Cells were seeded onto 25-cm^2^ T-flask and incubated at 37 °C in 5% CO2 atmosphere. After the first week of culture NSCs began to form neurospheres. At 2-day intervals, neurospheres were collected and passaged by a gentle enzymatic and mechanical dissociation.

Transient transfection was performed in NSCs dissociated and kept in proliferation medium by reverse transfection by overlaying DNA-lipofectamine complexes (lipofectamine 2000 Invitrogen) containing a GFP- or a GFP-Nup153 vector (Origene). Two days after transfection neurospheres were dissociated and cells were plated on matrigel-coated chamber slide for immunofluorescence or harvested for biochemical analysis. Each experiment was visually checked for transfection efficiency by analyzing GFP signal. In proteomics experiments, neurospheres cultures were dissociated, plated as single cells onto Matrigel Matrix (Becton Dickinson, Franklin Lakes, NJ) pre-coated Petri dishes and let to proliferate for 48 h.

### Animals and treatments

3×Tg-AD (*B6;129-Psen*^tm1Mpm^*Tg /APPSwe, tauP301L/1Lfa/Mmjax*) mice, expressing APP, PSEN and tau mutated proteins, were used as animal model of AD. In this study, 3×Tg-AD adult (7-month-old) mice and 0-1-day-old pups, used for NSC isolation (5–6 pups for each preparation) were compared with age-matched WT control mice (B6129SF2/J). All mice were randomly assigned to receive Nup153 or GFP-control lentiviral particles (1.5 µl; 1 × 10^9^/ml Origene CW305175V: lentiviral particles produced from PS100071 and MR211997L4) that were delivered in the hippocampus by a stereotaxic injection. Specifically, animals were deeply anesthetized (Ketamine 65 mg/Kg + Medetomidine 0.65 mg/Kg) and placed on a stereotaxic apparatus. For viral injections the following coordinates directed at the dorsal dentate gyrus were used: -2.1 mm posterior to bregma; ±1.6 mm lateral to midline and − 2 mm dorsoventral from the dura. The bone was removed using a drill and an injector cannula connected to a polyethylene tube filled with viral solution was slowly lowered for the injection. Flow rate (0.5 µL/min) was controlled through a syringe attached to the polyethylene tube and connected to an automatic pump. A total volume of 1.5 µL was injected in each site. After the injection, the injector was slowly retracted to avoid spilling of the viral solution. At the end of the procedure, the skin of the animals was sutured and disinfected, and animals were placed on a heating pad for recovery. Ten days after vector delivery by intra-hippocampal injection, mice were injected with BrdU (i.p. 50 mg/Kg) for five consecutive days and then formalin perfused for immunohistochemical analysis 2 h after the last BrdU injection or after 1 month for analysis of early and late marker of neurogenesis respectively. Cognitive test was performed 1 month after virus delivery. All procedures were performed in agreement with the ARRIVE guidelines 2.0.

### Behavioral test

Spatial learning and memory were evaluated by the Morris Water maze test (MWM) [4], Mice were trained to find a platform hidden 1 cm below the surface of a pool filled with water made opaque with white non-toxic paint. The acquisition training session started 4 days before the test session (probe test) and consisted of six trials a day for four consecutive days, during which each mouse was allowed to reach the platform within 40 s. Starting points were changed for each trial. A trial lasted either until the mouse found the platform or for a maximum of 40 s. Mice failing to find the platform were guided by the experimenter to the platform location and allowed to stay for 10s. The time taken to reach the platform (latency) were recorded with an automated video tracking system (ANY-maze™, Stoelting Co., IL). The probe test session was performed 24 h after the last day of the training. In this session the platform was removed from the pool and each mouse was allowed to swim for 60 s. Time spent in each quadrant was measured and the time of each quadrant was compared to time spent in the target quadrant.

### Superoxide anion measure

Oxidative stress was evaluated by measuring the level of intracellular superoxide anion by the fluorescent probe Dihydroethidium (DHE, Invitrogen Molecular Probes). NSCs were plated at a density of 150,000 cells/chamber glass, previously coated with Matrigel (Becton Dickinson, Franklin Lakes, NJ) and the day after were incubated with DHE (10 µM) in PBS at 37 °C for 30 min in the dark. After two washes in PBS, cells were fixed with 4% paraformaldehyde for 10 min. DHE fluorescence was recorded by a confocal laser scanning microscope (Nikon-Ti Eclipse) and 3D images of cells, deriving from a reconstruction of z series, were used for the analysis performed with Image J software. Signals from single ROIs (200–300 cells/each biological replicate) were used to calculate the average values (mean) for fluorescence intensity (MFI).

### Confocal analysis

Mice were anesthetized with a cocktail of ketamine (100 mg/mL) and xylazine (1 mg/mL) and transcardially perfused with saline solution followed by 4% paraformaldehyde. Brains were removed, post-fixed overnight at 4 °C, transferred in a solution of 30% sucrose for two days and then frozen in OCT. Coronal brain sections were cut with a cryostat (40-µm-thick) and processed for immunofluorescence. Free-floating sections were washed with Phosphate buffer (PB) 0.1 M for 10 min and then incubated with pre-heated HCl 2 N for 30 min at 37 °C under agitation, to denature DNA. HCl was neutralized with PB 0.1 M for 10 min in agitation. Sections were blocked with blocking solution (Triton 0.5%, BSA 5%, NGS 3%) for 60 min, and then incubated with anti-BrdU (1:400, rat Abcam Ab6326) primary antibody diluted in blocking solution (1:2) for 48 h at 4 °C on a shaker. After rinsing twice with PB 0.1 M for 5 min, sections were incubated with secondary antibody (anti-rat 1:300 CY3-conjugated) in BSA 5% for 90 min. After washing, a second immunofluorescence for DCX (1:300, Cell signalling 4604), PSA-NCAM (1:300, Millipore MAB5324) or Neu-N (1:300 Millipore MAB377) was performed at 4 °C for 48 h in agitation followed by washing and secondary antibody (anti-rabbit or anti-mouse 1:300 CY5-conjugated) in BSA 5% for 90 min. Sections were mounted with prolong gold antifade and analyzed by confocal analysis (Nikon-Ti Eclipse equipped with a 20x-40x-63x-100x objective). The number of GFP-, DCX-, PSA-NCAM- or NeuN-positive cells alone or in combination with BrdU was determined on z-stack reconstruction images in 40 μm-thick serial sections at 240 μm rostrocaudal intervals from the hippocampus. Positive cells were counted in the entire GCL-SGZ region with a 40x or 63× oil immersion objectives. Characterization of iPSCs was performed by detection by immunofluorescence and western blot of the following stemness markers: TRA1, Sox2, Nanog, SSEA-4, Oct4 with the StemLight™ Pluripotency Antibody Kit (1:1000 WB, 1:200 IF, Abcam 9656).

### Proteasome assay

Proteasome activity was measured in NSCs homogenized in lysis buffer containing: 50 mM HEPES (pH 7.5), 5 mM EDTA, 150 mM NaCl, and 1% Triton X-100. 100 µg of total proteins were run in duplicate in an ELISA-based assay according to manufacturer’s instructions (20 S proteasome activity assay, Chemicon).

### Proteomic analysis

NSCs isolated from WT and 3xTg mice were lysed in buffer containing: 50 mM Tris-HCl (pH 7.4), 250 mM NaCl, 0.1% tritonX100, 5mM EDTA, 0.3% empigen BB and supplemented with 1 mM PMSF and protease inhibitor mix. Protein extracts (1.5 mg for WT-NSCs and 3 mg from AD-NSCs) were immunoprecipitated using anti-Nup153 antibody (IP 1:30, Abcam ab93310) in IP-buffer (20 mM Tris HCl pH 8, 150 mM NaCl, 10% glycerol, 0.5% Nonidet P-40 (NP-40), 1 mM EDTA) using antibody conjugated with Ademtech´s Bio-Adembeads paramagnetic beads overnight. Different amounts of total protein were used to balance the different expression levels of Nup153 present in the two mouse strains in order to have comparable level of bait protein in IP. Eluates from IP were processed at Cogentech services (Milan). Briefly, samples were run on SDS-page gel and lanes were excised from the gel, reduced, alchylated and digested with trypsin before processing with the nLC-ESI-MS/MS QExactive-HF system.

All MS/MS samples were analyzed using Mascot (Matrix Science, London, UK; version 2.6.0). Mascot was set up to search the CP_Mouse_2020_20200706 database. Mascot was searched with a fragment ion mass tolerance of 0.020 Da and a parent ion tolerance of 10.0 PPM. Oxidation of methionine and acetyl of the N-terminus were specified in Mascot as variable modifications.

CRITERIA FOR PROTEIN IDENTIFICATION Scaffold (version Scaffold_4.11.0, Proteome Software Inc., Portland, OR) was used to validate MS/MS based peptide and protein identifications. Peptide identifications were accepted if they could be established at greater than 95.0% probability by the Scaffold Local FDR algorithm. Protein identifications were accepted if they could be established at greater than 99.0% probability and contained at least 2 identified peptides. Protein probabilities were assigned by the Protein Prophet algorithm [26]. Proteins sharing significant peptide evidence were grouped into clusters.

### Functional enrichment analysis and network construction

Molecular processes and disease pathways were analyzed on the basis of protein-protein interaction revealed by proteomics. We performed functional enrichment analysis by querying GO Gene Ontology [27] and the Kyoto Encyclopedia of Genes and Genomes (KEGG) [28] though Enrichr web tool [29]. *P*-values were adjusted with the Benjamini–Hochberg method and a threshold equal to 0.05 was set to identify functional annotations significantly enriched amongst genes of the given input list. Networks were constructed and analyzed though Cytoscape (v3.8.2) open source software [30].

### Western blot

NSCs or brain organoids were lysed in RIPA buffer (10 mM Tris-HCl (pH 7.4), 140 mM NaCl, 1% tritonX100, 0.1% sodium deoxycholate, 0.1% SDS, 1 mM EDTA) plus 1 mM PMSF and protease inhibitor mix. Standard Western blotting techniques were used, and the Uvitec imaging equipment was used to collect and interpret ECL signals. Optical density values of the specific proteins were normalized to that of beta-actin which in our experimental settings were unchanged. Results are expressed as fold change versus wild type/control samples, which were considered equal to 1. Representative WBs are shown in the figures and graphs show the mean of at least three independent experiments ± standard error of the mean (SEM). The following antibodies were used: anti-Nup153 (1:500, Abcam Ab12172), anti-DCX (1:200, Cell signalling 4604), anti-βIII tubulin (1:300, Abcam ab18207), anti-MAP2 (1:1000, Sigma M9942), anti-NF (1:1000, SIGMA N4142), anti-NeuN (1:1000, Millipore MAB377), anti-HDAC4 (1:1000, Abcam Ab12172), anti-CTCF (1:1000, BETHYL BLR041F), anti-Sox2 (1:1000, Abcam Ab97959), anti-TP63 (1:1000, Sigma ZRB1754-25UL), anti-Brd4 (1:1000, Sigma ZRB1693), anti-H3K14ac (1:1000, Abcam Ab52946), anti-H3K9ac (1:1000, Abcam Ab12179), anti-H4K20me3, (1:1000, Abcam Ab9053), anti-Nup50 (1:1000, Abcam Ab85915), anti-beta-actin (1:2000, Abcam ab8227). Dot blot experiments were performed using 5 µl (15 µg of proteins) of lysate spotted directly on the membrane. After blocking (milk 5% in TBST), membranes were probed with the anti-Aβ (1:1000, BioLegend 803014-6E10), anti-nitro-tyrosine (1:1000, Abcam Ab42789) anti-nitroso-S-cysteine (1:1000, Alpha Diagnostic NISC11A) o.n. and then the signal revealed by secondary antibody and ECL reaction as in western blot. Optical density values of dot blot signals were normalized to red ponceau (RP) staining to verify total protein loading. Results are expressed as fold change versus control samples, which were considered equal to 1.

### iPSC generation

iPSCs were produced from skin biopsies of sporadic AD patients (*n* = 3, age range 62–68, male). Enrolled patients fulfilled the clinical criteria for AD according to the National Institute on Aging-Alzheimer’s Association workgroups: cognitive impairment documented with a formal and extensive neuropsychological evaluation; positive biomarkers indicating the AD pathophysiological process (PET amyloid imaging and structural magnetic resonance imaging evidencing disproportionate atrophy in medial, basal and lateral temporal lobe and medial parietal cortex). Control biopsies were obtained from PET-negative patients, matched for gender and age, enrolled for non-neurological disorders (*n* = 3). iPSCs were generated from fibroblast by a non-integrative method for reprogramming human cells based on a modified non-transmissible form of Sendai virus that does not integrate into the host genome or alters the genetic information of the host cell. iPSCs were cultured in Stemflex medium (Thermo Fisher) for 1 month.

### Brain organoids culture

iPSC colonies were dissociated into single cells with the Gentle Cell Dissociation Reagent (STEMCELL technologies) and plated onto matrigel-coated 6 well plate at a density of 400,000 cells/ml. The day after, the cells were infected with the LV-EF1a-GFP or the LV-EF1a-Nup153 lentiviral vectors (MOI 5) in StemFlex medium plus polybrene (8 µg/ml) and ROCK inhibitor (10µM). Nup153 DNA from MR211997L4 plasmid was cloned by the Precision Shuttle System (Origene) in the pLenti-EF1a-C-mGFP plasmid and lentiviral particles containing GFP and Nup153 were produced by standard procedures in HEK293T cells. To increase the efficiency the plate was centrifuged at 1000 g for 1 h at 25 °C. The day after the medium was replaced (StemFlex) and two days later the iPSCs were used to produce brain cortical organoids (COs) by the STEMdiff™ Cerebral Organoid Kit (STEMCELL technologies). Briefly, at day 0 hiPSC colonies were dissociated into single cells and 9000 cells/well were plated in ultra-low attachment 96-well plates (Corning Costar) in EB Formation Medium supplemented with 10 µM Y-27,632 (STEMCELL technologies). The plate was placed at 37 °C with 5% CO2 without disturbing it for 24 h. At day 2 and 4, EB Formation medium was added to the culture. At day 5, embryoid bodies were transferred to a 24-well ultra-low attachment plate (Corning Costar) containing Induction Medium. At day 7, embryoid bodies were embedded into Matrigel droplets and placed in Expansion Medium in a 6-well ultra-low attachment plate (Corning Costar). At day 10, organoids were cultured in maturation medium, replaced every 2–3 days, and analyzed at 1 and 2 months. For immunofluorescence analysis COs were fixed in 4% paraformaldehyde for 24 h and then transferred in a solution of 30% sucrose for one day and then frozen in OCT. Cryosections were processed with the following antibodies: anti-Sox2 (1:200, Cell signalling, #4604), anti-MAP2 (1:300, Sigma M9942), anti-synapsin I (1:500, Abcam Ab254349), anti-PSD95 (1:300, Cell Signalling #3550), anti-N-Cadherin (1:300, Abcam Ab18203), anti ZO-1 (1:300, Abcam Ab59720), anti-Nav1.6 (1:300, Sigma WH0006334M4).

### Statistics

Sigmaplot 14.0 software was used to calculate significance. Sample size (n) is indicated in the main text and represents independent experiments from different cell culture preparations or animals. Comparison of two groups was performed by t-test. Multiple comparisons were performed by One-Way Analysis of Variance (ANOVA) followed by Bonferroni post hoc test for normally distributed samples or ANOVA on RANKS followed by post hoc tests for non-normally distributed samples. *P* values < 0.05 were considered as significant in all tests. For all analyses, the observer was blind to the identity of the samples.

## Results

### Proteomic-based approach reveals the implication of Nup153-protein networks in neurodegenerative, oxidative and degradative pathways in NSCs from AD mice

**Fig. 1:**
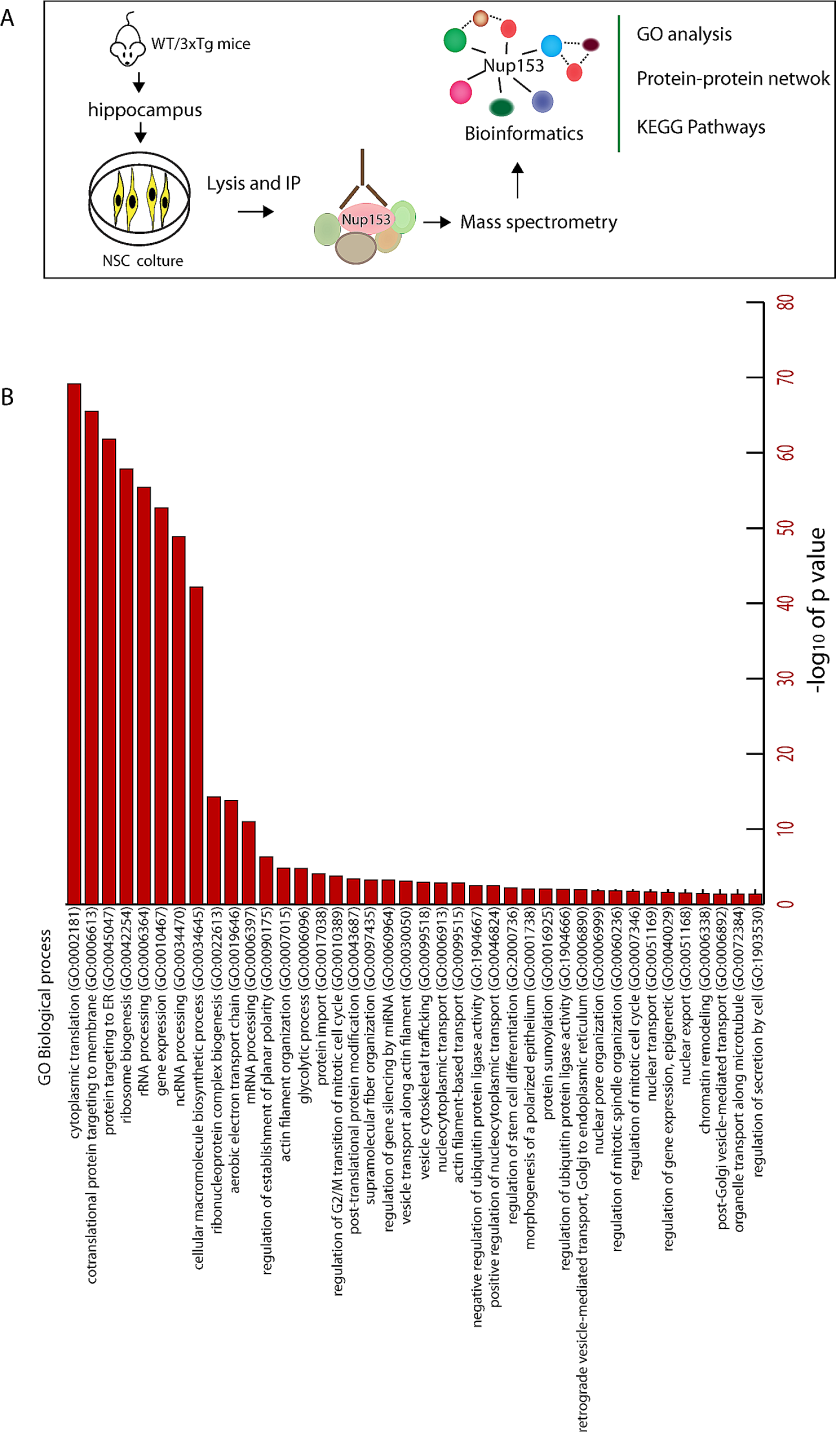
(**A**) Cartoon depicting the experimental plan for the in vitro experiments. (**B**) Schematic illustrating the main biological processes from Gene ontology analysis of Nup153-protein network in WT-NSCs

A proteomic approach was applied to identify the molecular partners of Nup153 that can be altered in AD and contribute to the dysfunction of AD-NSCs. To this aim, Nup153 was immunoprecipitated from AD- and WT-NSC extracts and eluates were analyzed by mass spectrometry with the in-gel digestion method, called STAGE-diging. We previously found that Nup153 protein levels in AD-NSCs are 50% lower than in WT-NSCs [25], so we processed different amounts of proteins in IP to balance the Nup153 level in the two genotypes and start with the same amount of bait (Nup153: 1.5 mg WT-NSCs; 3.0 mg AD-NSCs). Although the proteomic analysis identified the same number of peptides for the bait Nup153 in WT- and AD-NSCs, indicating that in the two conditions we immunoprecipitated the same amount of Nup153, we proceeded with a qualitative analysis to uncover changes in the quality of interaction between normal and disease conditions. Gene ontology (GO) enrichment analysis based on protein-protein interaction revealed by proteomics was used to highlight the molecular and disease pathways involved (see experimental plan in Fig. 1A).

We identified a total of 701 proteins that co-immunoprecipitated with Nup153 in WT-NSCs (Suppl. Figure 1). GO analysis of molecular processes indicated that Nup153 was involved in many pathways including: (1) nuclear-cytoplasmic transport of several cellular components including the export of proteins, tRNA, non-coding RNA, mRNA; (2) ribosome biogenesis, rRNA and ncRNA processing; (3) regulation of gene expression and protein-DNA complex assembly, chromatin remodeling; (4) regulation of translation; (5) protein targeting to membrane, ER and mitochondria; (6) energy metabolism and regulation of biosynthetic pathways; (7) regulation of mitosis, cell spindle reorganization and localization, nuclear pore organization and nuclear membrane reassembly; (8) regulation of stem cell differentiation and establishment of epithelial polarity and differentiation; (9) regulation of protein stability and degradation via ubiquitin protein ligase activity and SUMOylation; (10) actin reorganization and vesicle trafficking (Fig. 1B).

To uncover possible differences in the Nup153 protein network we qualitatively analyzed the Nup153-bound proteins in WT- and AD-NSCs. We found 165 and 159 specific Nup153 interactors in WT- and AD-NSCs, respectively (Suppl. Figure 1).

GO analysis of biological processes (BP) showed that in WT-NSCs the Nup153 network was centered on RNA metabolism, epigenetic mechanisms and transport (tRNA, mRNA and ncRNA regulation and transport; ribosome biogenesis; miRNA regulation; chromatin remodeling) (Suppl. Figure 2 A). Evaluation of Nup153-bound proteins in AD-NSCs showed, in addition to RNA-based mechanisms, an enrichment of proteins involved in energy processes and mitochondrial function (Suppl. Figure 2B). To further explore the relevant molecular interaction/reaction network in AD-NSCs, these data were analyzed by the KEGG database (a pathway maps based on the systematic analysis of molecular interaction, reaction and relation networks). We found the involvement of pathways of neurodegeneration and AD, mitochondrial dysfunction, proteasomal and catabolic processing, metabolism, cell cycle and RNA degradation (Suppl. Figure 3). These data suggest that the complex regulatory network orchestrated by Nup153 is based on multiple interactions that are differently regulated in WT- and AD-NSCs.

### Nup153 overexpression normalizes proteasomal and oxidative stress pathways in AD-NSCs

**Fig. 2:**
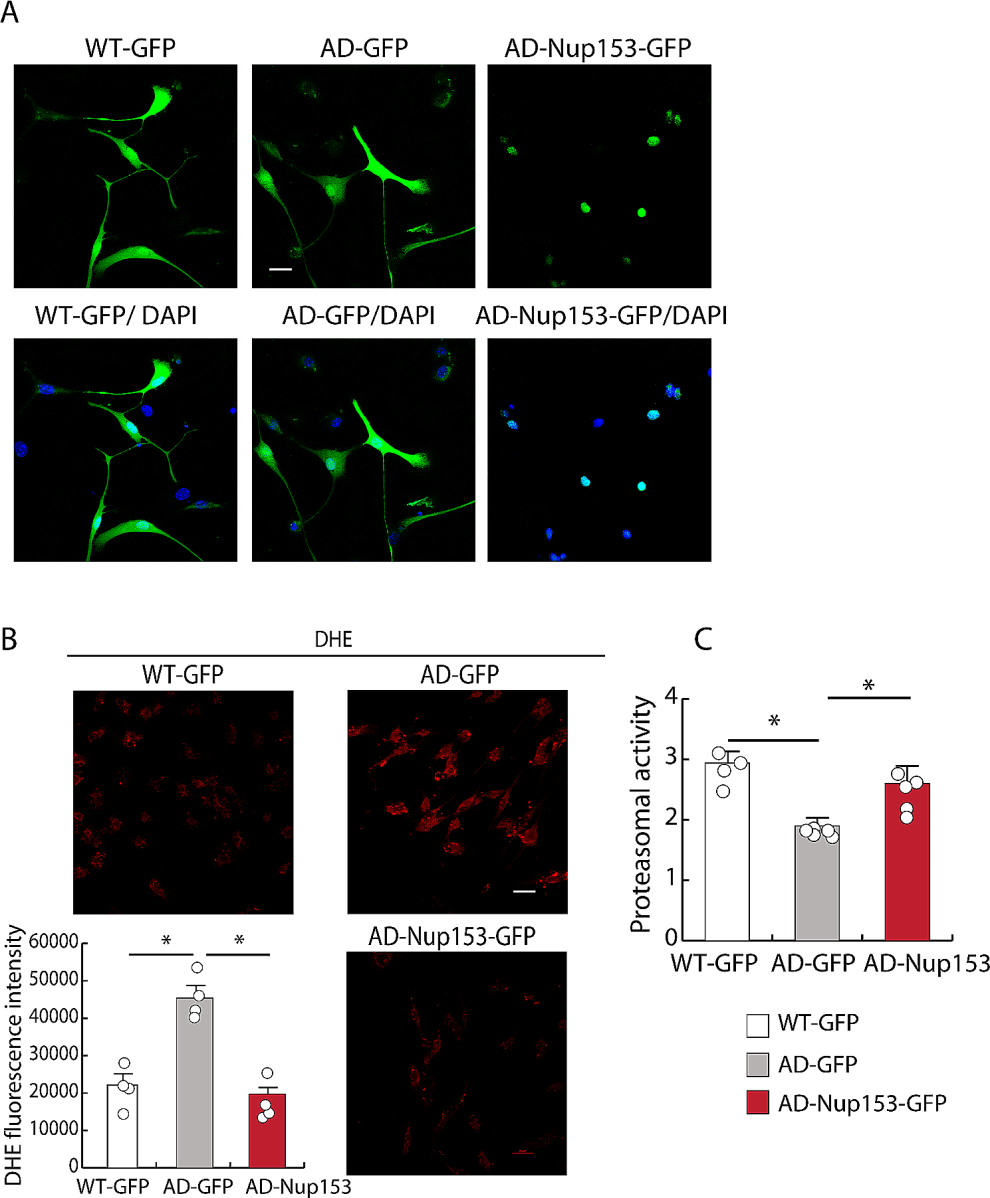
Nup153 overexpression in AD-NSCs counteracts oxidative stress and recovers proteasomal activity. (**A**) Immunofluorescence showing GFP expression in AD-NSCs transfected with GFP or GFP-Nup153 vectors at 72 h. (**B**) DHE labelling of NSCs in the above conditions to reveal the level of oxidative stress. The graph indicates the mean fluorescence intensity for DHE (*n* = 4). (**C**) Proteasomal activity measured in WT-, AD-GFP- and AD-GFP-Nup153-NSCs (*n* = 5). *P < 0.05. Scale bar 20 μm

Proteomic analysis revealed that Nup153 protein network in AD-NSCs is associated with changes in several pathways. Since proteasomal degradation and oxidative stress are reported as two key contributors to AD pathology for their role in Aβ and tau accumulation [25] we focused on these pathways to further validate Nup153 function. Thus, because Nup153 levels in AD-NSCs are lower than in WT-NSCs, we investigated whether restoring appropriate levels of this protein could impact oxidative stress and proteasomal degradation. AD-NSCs were transiently transfected with GFP or Nup153-GFP vectors. Whole-cell (WT-GFP, AD-GFP) or nuclear restricted (AD-Nup-GFP) GFP fluorescence confirmed cell transfection and Nup153 re-expression as assessed by confocal microscopy. WT-GFP transfected NSCs were used as comparison (Fig. 2A). Oxidative stress was assessed by labeling WT- and AD-NSCs, expressing either GFP or Nup153, with DHE, a red sensor for superoxide and hydrogen peroxide species. We found that the mean fluorescence intensity (MFI) for DHE was higher in AD-NSCs compared with WT-NSCs, but it was reduced in AD-NSCs overexpressing Nup153 (DHE MFI: WT-NSC-GFP 22,173 ± 3,096; AD-NSC-GFP 45,351 ± 3,316; AD-NSC-GFP-Nup153 19,713 ± 2,047, ANOVA with Bonferroni test *P* < 0.001, *n* = 4; Fig. 2B).

Analysis of proteasomal function in the above conditions showed that activity was lower in AD-NSCs compared with WT-NSCs. However, the presence of Nup153 in AD-NSCs increased proteasomal activity (activity [µM]: WT-NSC-GFP 2.94 ± 0.12; AD-NSC-GFP 1.89 ± 0.01; AD-NSC-GFP-Nup153 2.50 ± 0.136 ANOVA with Bonferroni test *P* < 0.001, *P* < 0.05, *n* = 4–5; Fig. 2C).

### Nup153 overexpression in the hippocampus improves neurogenesis and cognitive function in the 3×Tg mouse model of AD

**Fig. 3:**
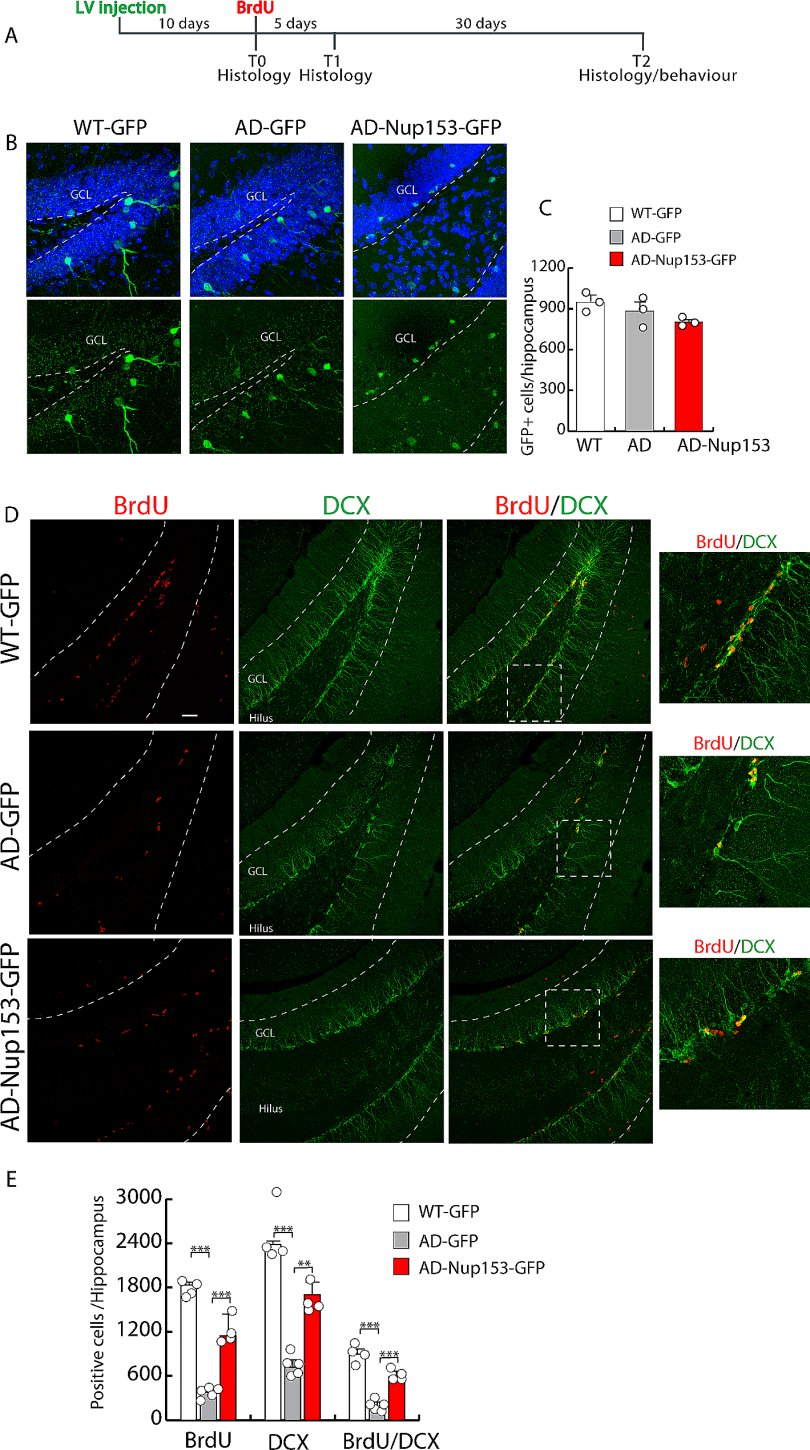
Nup153 overexpression in the hippocampus of AD mice stimulates proliferation and early differentiation. (**A**) Experimental plan for the in vivo experiments. (**B**) Representative confocal images showing GFP positive cells in the hippocampal niche of WT and AD mice injected with a lentivirus coding for GFP (WT and AD) or GFP-Nup153 at 10 days (T0). (**C**) Quantification of the GFP + cells in WT-GFP, AD-GFP and AD-GFP-Nup153 injected mice. Nuclei were counterstained with DAPI (*n* = 3, scale bar 20 μm). (**D**) Representative confocal images showing hippocampal cells positive for BrdU (red) and DCX (green) from WT-GFP, AD-GFP and AD-GFP-Nup153 injected mice at T1. Arrows indicate BrdU/DCX double positive cells. (**E**) The graph shows the number of BrdU^+^, DCX^+^ and BrdU/DCX double positive cells in WT-GFP, AD-GFP and AD-GFP-Nup153 injected mice (*n* = 4, scale bar 50 μm). GCL = granule cell layer, ***P < 0.001, **P < 0.01

Results of our in vitro experiments suggest that Nup153 is involved in many NSC pathways. To assess its role in in vivo postnatal neurogenesis, we overexpressed Nup153 in the hippocampal neurogenic niche of AD mice by mean of a lentiviral vector (AD-Nup153) (see experimental plan Fig. 3A). WT and AD mice injected with a GFP-empty vector were used as controls (AD-GFP; WT-GFP). Efficiency of vector delivery was checked by immunofluorescence analysis of GFP^+^ cells in the DG of WT-GFP, AD-GFP and AD-Nup injected mice at 10 days (T0) (Fig. 3B-C). To assess neurogenesis, 10 days after vector delivery, mice were injected with BrdU (50 mg/Kg, i.p.) for five consecutive days and then analyzed immediately (T1) or after 1 month (T2) by confocal microscopy.

**Fig. 4:**
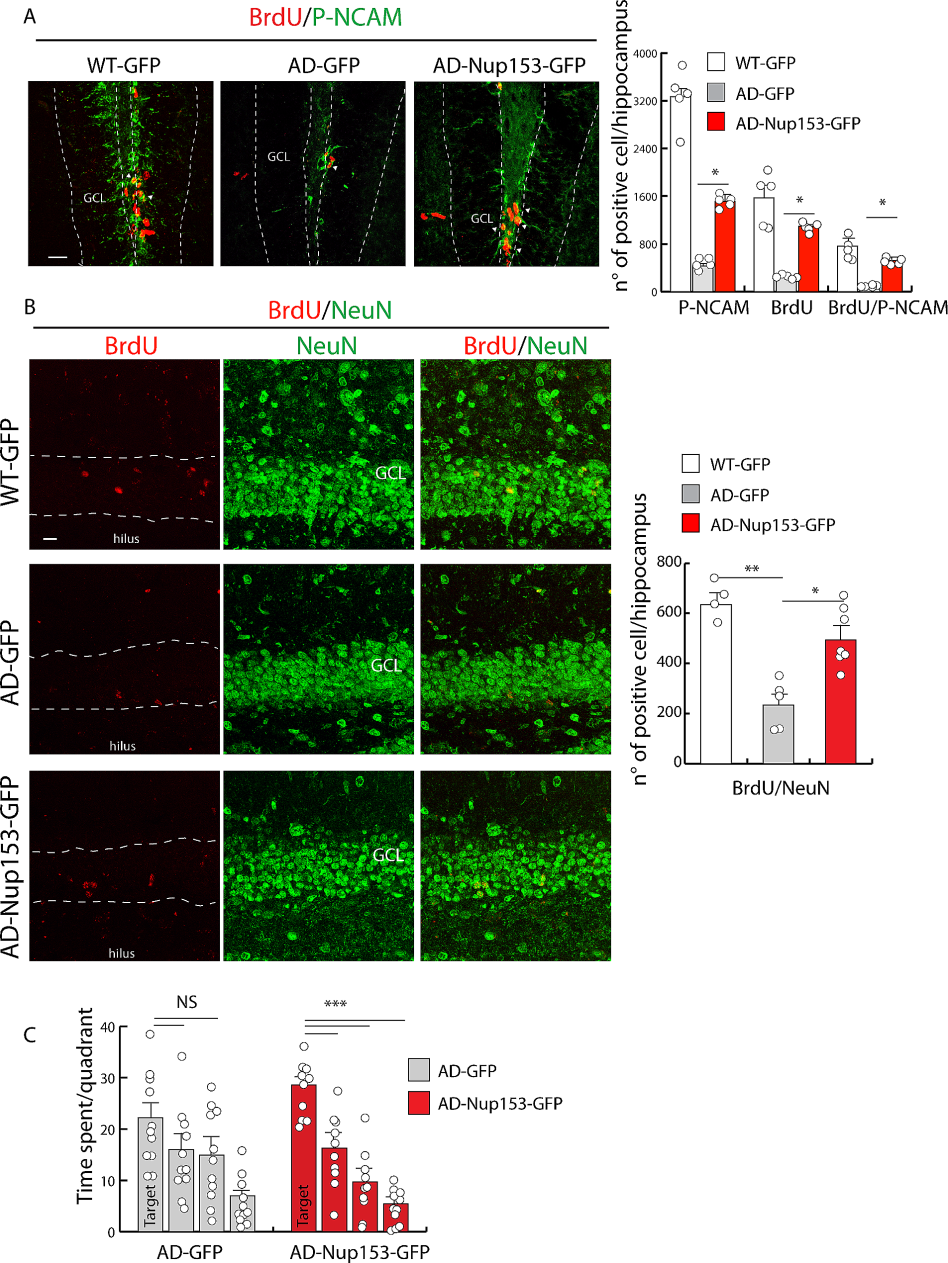
Nup153 overexpression in the hippocampus of AD mice promotes neurogenesis and cognitive performance. (**A**) Representative confocal images showing hippocampal cells positive for BrdU (red) and P-NCAM (green) from WT-GFP, AD-GFP and AD-GFP-Nup153 injected mice at T1. Arrows indicate BrdU/P-NCAM positive cells. The graph shows the number of BrdU^+^, P-NCAM^+^ and BrdU/P-NCAM double positive cells in the above conditions, *n* = 5 scale bar 20 μm. (**B**) Representative confocal images showing BrdU^+^ cells (red) in the granule layer of the hippocampus identified by NeuN^+^ cells in WT-GFP, AD-GFP and AD-GFP-Nup153 injected mice at T2 (scale bar 10 μm). The graph shows the number of BrdU/NeuN double positive cells in the hippocampus in the above conditions (*n* = 4–7). (**C**) Assessment of memory performance by the Morris Water Maze test (MWM). The graph shows the time spent in the four quadrants during the probe test performed on day 5 of MWM. *P < 0.05, **P < 0.01, ***P < 0.001

Early induction of neurogenesis was assayed by comparative evaluation of proliferation by BrdU incorporation and co-expression of the doublecortin (DCX) in the three groups. Compared with AD-GFP mice, Nup153 overexpression in AD mice greatly increased the number of proliferating cells (BrdU^+^: WT-GFP 1,838.6 ± 42.0, AD-GFP 381.5 ± 23.0, AD-Nup153-GFP 1,055.0 ± 114.0, *P* < 0.001 ANOVA with Bonferroni post-hoc test, *n* = 4–5), the number of early differentiating cells only positive for DCX (DCX^+^: WT-GFP 2615.2 ± 208, AD-GFP 716.7 ± 61, AD-Nup153-GFP 1599.85 ± 101, *P* < 0.05 ANOVA with Bonferroni test, *n* = 4–5) and those double positive for BrdU and DCX (BrdU^+^/DCX^+^: WT-GFP 888.30 ± 55, AD-GFP 177 ± 28, AD-Nup153-GFP 565.6 ± 33, *P* < 0.001, *P* < 0.01, ANOVA with Bonferroni post-hoc test, *n* = 4–5) (Fig. 3D-E**).** We previously demonstrated that cultured AD-NSCs, compared with WT-NSCs, express lower level of the polysialylated form of NCAM (P-NCAM), which plays an important role in neural cell migration and invasion. Of note, Nup153 overexpression recovered P-NCAM level and migration of AD-NSCs in vitro [25]. Thus, we analyzed the numbers of BrdU^+^/P-NCAM^+^ cells in Nup153 injected mice compared to WT and AD control mice. Figure 4 shows that the number of cells positive for BrdU, P-NCAM and BrdU/P-NCAM in the hippocampal niche of AD-GFP mice is lower compared with WT-GFP mice but it is partially recovered in AD-Nup153-GFP mice (P-NCAM^+^: WT-GFP 3268 ± 215, AD-GFP 428 ± 22, AD-Nup153-GFP 1512 ± 37; BrdU^+^: WT-GFP 1572 ± 220, AD-GFP 246 ± 9, AD-Nup153-GFP 1093 ± 30; BrdU^+^/P-NCAM^+^: WT-GFP 761 ± 82, AD-GFP 80 ± 4, AD-Nup153-GFP 518 ± 14, *P* < 0.05 ANOVA on RANKS followed by Student-Newman-Keuls Method, *n* = 5; Fig. 4A).

Differentiation of progenitors in more mature neurons and integration in the granule cell layer was evaluated one month after the last injection of BrdU by confocal analysis of BrdU^+^/NeuN^+^ double labelled cells. We found that the number of BrdU^+^/NeuN^+^ cells was lower in AD-GFP mice compared with WT-GFP mice but was increased in AD-Nup153-GFP mice (BrdU^+^/NeuN^+^: WT-GFP 635 ± 33, AD-GFP 234 ± 41, AD-Nup153-GFP 494 ± 46 *P* < 0.05 ANOVA followed by Bonferroni test, *n* = 4 WT, *n* = 5 AD, *n* = 7 AD-Nup153; Fig. 4B).

At the 1-month time-point, animals were tested for learning and spatial memory recovery by the Morris Water Maze (MWM). In the training phase, no statistically significant differences were observed between AD-GFP and AD-Nup153 mice. However, in the probe test, AD-Nup153 animals were able to discriminate the target quadrant with respect to other quadrants (*n* = 11, Student’s t-test, Target vs. Q1/Q2/Q3 *P* < 0.001), whereas GFP-AD mice failed to do it (*n* = 10, Student’s t-test, Target vs. Q1 *P* = 0.17; Target vs. Q2 *P* = 0.01; Target vs. Q3 *P* < 0.001; Fig. 4C).

### Nup153 overexpression ameliorates brain cortical development in AD-iPSC-derived organoids

To further investigate the role of Nup153 in NSC differentiation we took advantage of the developmental model of brain organoids derived from iPSCs. These pluripotent cells were generated from skin biopsies of sporadic AD patients (*n* = 3) and from healthy controls (*n* = 3) obtained from PET-negative patients, matched for gender and age. iPSC clones were characterized by confocal microscopy and western blot analyses for the following markers of pluripotency: TRA1, Sox2, Nanog, SSEA-4, Oct4 (Suppl. Figure 4 A-B). Among all these markers, Sox2 showed a decreased level in AD-iPSCs compared with control cells (WB optical density: C-iPSC 1 ± 0.02, AD-iPSC 0.54 ± 0.07, *P* < 0.01 t test, *n* = 3; Suppl. Figure 4B). In line with our previous data on AD-NSCs we found lower Nup153 levels in AD-iPSCs as assessed by confocal and western blot analyses (WB optical density: C-iPSC 1 ± 0.03, AD-iPSC 0.47 ± 0.08, *P* < 0.05 t test, *n* = 3; Suppl. Figure 4 C-D). Further, we evaluated the level of histone modifications and those of chromatin architecture and reprogramming regulators (HDAC4, CTCF, Nup50, TP63, Brd4), known to interact with Nup153 and to contribute to gene regulation [24, 31] as indicators of chromatin status in C- and AD-iPSCs. The expression of HDAC4, CTCF and Nup50 was unchanged while that of TP63 was found lower in AD-iPSC compared with control cells (WB optical density: C-iPSC 1 ± 0.04, AD-iPSC 0.42 ± 0.06, *P* < 0.01 t-test, *n* = 3; Suppl. Figure 5 A-B). Confocal analysis revealed decreased levels also of Brd4 in AD-iPSC (MFI: C-iPSC 27460.85 ± 913; AD-iPSC 10683.68 ± 384, Mann-Whitney t test *P* < 0.001, *n* = 70–75 nuclei, Suppl. Figure 5 C). Assessment of histone marks showed the presence of increased levels of acetylated histone H3 on lysine 14 (H3K14ac) and lysine 9 (H3K9ac), while tri-methylation on histone H4 lysine 20 (H4K20me3) levels were unchanged in AD-iPSCs in comparison with C-iPSCs (WB optical density K14ac: C-iPSC 1 ± 0.01, AD-iPSC 2.03 ± 0.30 *P* < 0.05; K9ac: C-iPSC 1 ± 0.04, AD-iPSC 1.92 ± 0178 *P* < 0.01; K20me3: C-iPSC 1 ± 0.04, AD-iPSC 0.98 ± 0.03 t-test, *n* = 3 Suppl. Figure 5 A-B).

**Fig. 5:**
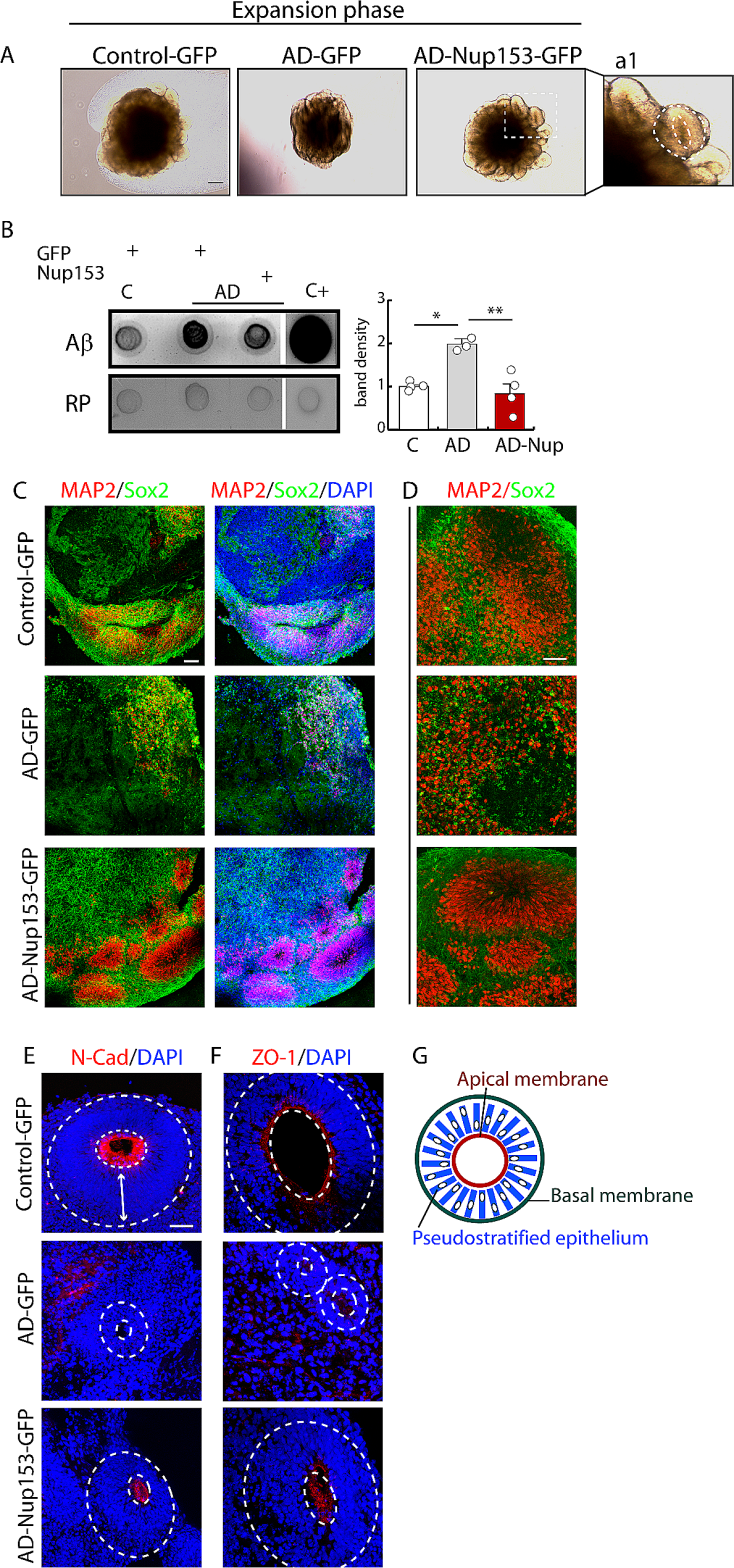
Nup153 transduction in AD-iPSCs improves the maturation and organization of brain cortical organoids. (**A**) Phase contrast representative images of control, AD and AD-Nup153 organoids in the expansion phase. a1) Enlargement showing a detail of the circular neuroepithelium-like structure surrounded by the apical and basal membranes indicated by the dotted lines. (**B**) Aβ levels by dot blot analysis (*n* = 3–4). Each lysate was obtained from the pool of 3–4 individual organoids. Hippocampal lysate from 9-month-old 3×Tg mice was used as positive control. Red ponceau (RP) staining was used as loading index and used to normalize samples. C-F) Confocal analysis of MAP2/Sox2 (**C** and **D** at different magnification) and N-Cadherin (**E**) and ZO-1 (**F**) in control, AD and AD-Nup153 brain organoids. Nuclei were counterstained with DAPI, scale bar 50 μm. The inner dotted white circles indicate the ring-like N-cadherin and ZO-1 distribution at the apical membrane. The double arrow indicates the pseudostratified neuroepithelium starting from the cavity to the basal membrane. (**G**) Scheme representing the organization of the pseudostratified epithelium between the inner apical membrane and the outer basal membrane in the organoid. *P < 0.05, **P < 0.01

AD-iPSC cells were transduced with a Nup153 lentivirus and used for brain organoid production (Suppl. Figure 5D-E). Control cells were infected with a GFP-coding lentiviral vector. Under optical microscope evaluation, soon after neuroectoderm formation we observed a different growth pattern with fewer areas of expansion in AD-organoids compared with control ones (Fig. 5A). Conversely, organoids derived from Nup153-tranduced AD-iPSC (Nup-AD organoids) showed an expansion comparable with control ones and the formation of circular neuroepithelium-like structures (Fig. 5a1). At 1 month of maturation, organoids were either fixed for immunofluorescence or lysed for biochemical analysis. Dot blot assay revealed that Aβ accumulated in AD organoids compared with control ones while its level was decreased in Nup-AD organoids (Fig. 5B optical density: C-GFP 1 ± 0.05, AD-GFP 1.97 ± 0.06, AD-Nup153-GFP 0.83 ± 0.25 *P* < 0.05 ANOVA followed by Bonferroni test, *n* = 3–4). Our prior study (25) and proteome analysis reveal a link between Nup153 dysregulation and oxidative/nitrosative stress. So, we evaluated nitro-oxidative stress markers such as nitro-tyrosine (N-Tyr) and cysteine-S-nitrosylation (SNO-cys) in control and AD organoids, with and without Nup153 expression. AD organoids displayed elevated levels of both N-tyr and SNO-cys compared to control organoids, as determined by dot blot assays. Nup153 transduction, on the other hand, decreased the concentration of these nitrosative stress markers in AD organoids. (Suppl. Figure 6 A-B, optical density N-tyr: C-GFP 1 ± 0.17, AD-GFP 3.71 ± 0.96, AD-Nup153-GFP 0.87 ± 0.22 *P* < 0.05 ANOVA followed by Bonferroni test; SNO-cys: C-GFP 1 ± 0.19, AD-GFP 4.01 ± 1.42, AD-Nup153-GFP 0.70 ± 0.11 *P* < 0.05 ANOVA on Ranks followed by Student-Newman-Keuls test; *n* = 3).

Evaluation by confocal analysis of Sox2/MAP2 double labelled organoids (Sox2-CY3-red; MAP2-CY5-green) showed that control organoids were characterized by the formation of neuroepithelial loops, positive for the neural progenitor marker Sox2, that were surrounded by MAP2^+^, i.e., differentiated cells. Of Note, AD organoids were instead predominantly formed by areas of disorganized cells positive for Sox2 but with a weak expression for MAP2. Nup-AD organoids showed a structure very similar to control ones with the formation of multiple neural loops (Fig. 5C-D) and recovery of the cortical organization. These data are in agreement with proteomic findings that show the association of Nup153 with several proteins belonging to the following categories: establishment of apical/basal cell polarity (CDC42, FSCN1); establishment of planar cell polarity (PSMD12, CLTC, AP2A1, AP2B1, PSMA5, CDC42, PSMB6, PSMA6, PSMB4, PSMC, VANGL2, PSMC3, PSMD2, PSMD3, RAC1, PFN1, AP2M1), establishment of epithelial cell polarity (CDC42, CYTH3, MYO18A), migration (RAC1, CDC42) and cell adhesion (PCDHGC3, PSMC, VANGL2, PFN1, CDC42). Among these proteins we started to address the involvement of those forming adherent junctions (AJ) since they have been reported to preserve cortical architecture during development [32] by establishing radial glia polarity. We hypothesized that altered neuroepithelium formation and organization in neural tube-like structures, that we observed in AD organoids, could be related at least in part to AJ modifications. Confocal analysis of N-cadherin and ZO1 revealed that these proteins formed, in control organoids, ring structures at the apical membrane in contact with a pseudostratified neuroepithelium. Of note, these rings were not detected in AD organoids but they were re-expressed in Nup153-treated AD organoids (Fig. 5E-G).

**Fig. 6:**
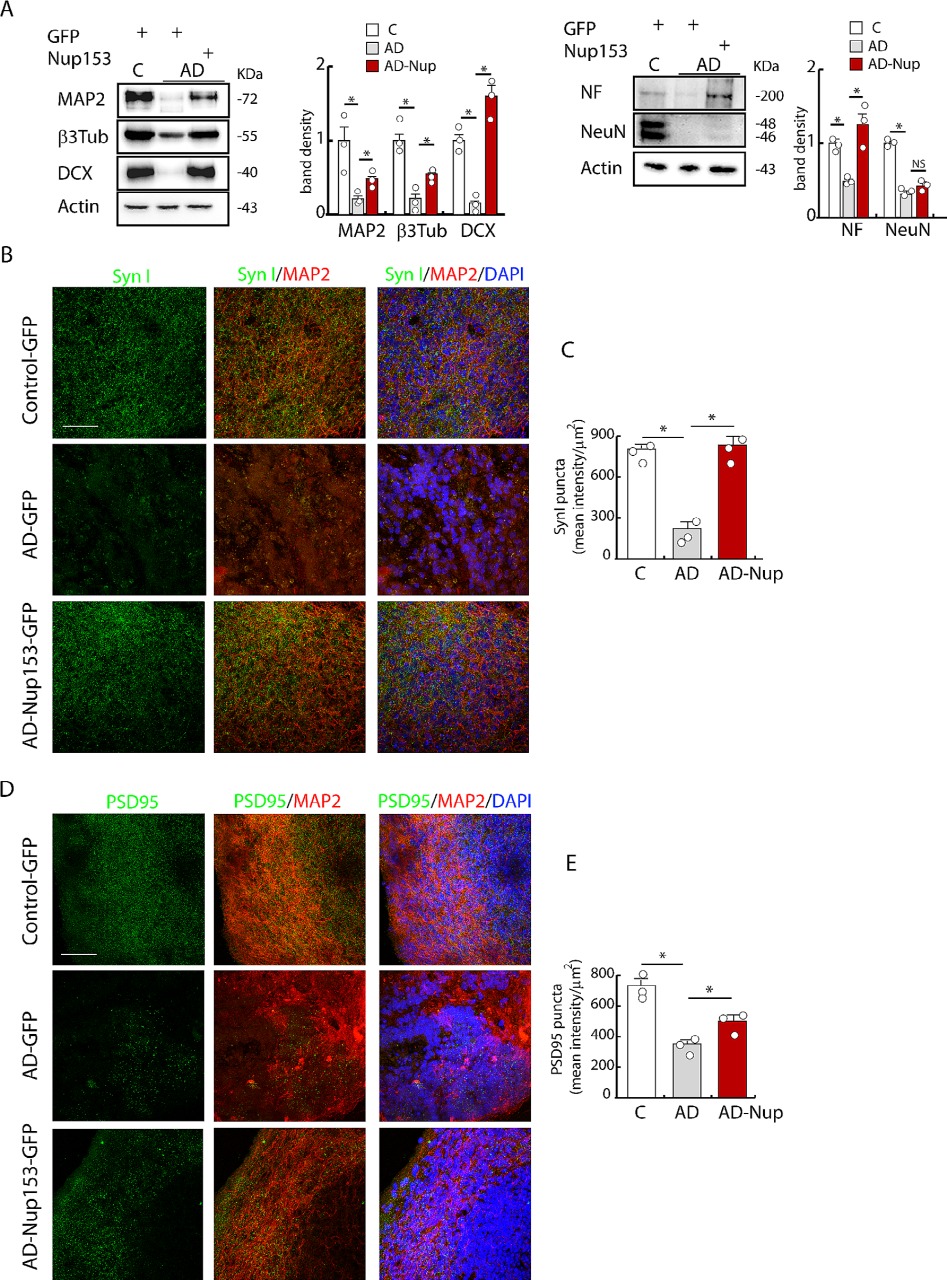
Nup153 transduction in AD-iPSCs improves the differentiation of brain cortical organoids. (**A**) Western blot analysis of MAP2, β3 tubulin, DCX, NeuN and NF proteins in brain organoids (*n* = 3) and relative quantification based on the expression of actin. Each lysate was obtained from the pool of 3–4 individual organoids. (**B**, **D**) Representative images of syn I and PSD95 expression from control, AD and AD-Nup153 brain organoids analyzed by confocal analysis and counterstained with MAP2 and DAPI (scale bar 50 μm) at 2 months of differentiation. (**C**, **E**) Quantification of mean fluorescence intensity of SynI and PSD95 puncta/area relative to control, AD and AD-Nup153 brain organoids (*n* = 3). *P < 0.05

At 2 months of maturation, western blot analysis confirmed that the developmental stage of control and AD organoids was different with higher expression of differentiation markers (MAP2, β3 tubulin, DCX, neurofilament (NF), NeuN) in control organoids compared with AD ones. Of note, the expression of these markers was partially recovered in Nup153-treated AD organoids (Fig. 6A, WB optical density: DCX: C-GFP 1 ± 0.09, AD-GFP 0.15 ± 0.07, AD-Nup153-GFP 1.59 ± 0.14; β3Tub: C-GFP 1.00 ± 0.13, AD-GFP 0.22 ± 0.08, AD-Nup153-GFP 0.54 ± 0.03; MAP2: C-GFP 1.00 ± 0.31, AD-GFP 0.21 ± 0.08, AD-Nup153-GFP 0.48 ± 0.02; *P* < 0.05 ANOVA on RANKs followed by SKN test, *n* = 3; NF: C-GFP 1 ± 0.04, AD-GFP 0.49 ± 0.17, AD-Nup153-GFP 1.25 ± 0.17 *P* < 0.05 ANOVA followed by Bonferroni test, *n* = 3). The level of NeuN in AD-Nup organoids showed a trend although not statistically significant (NeuN: C-GFP 1 ± 0.02, AD-GFP 0.31 ± 0.02, AD-Nup153-GFP 0.42 ± 0.04, *P* < 0.05 ANOVA followed by Bonferroni test, *n* = 3). Other mature pre- and postsynaptic markers such as synapsin I and PSD95 were analyzed by confocal microscopy and their levels were increased in AD after Nup153 transduction (Fig. 6B-E: SynI puncta: C-GFP 803.75 ± 28.79, AD-GFP 222.36 ± 0.08, AD-Nup153-GFP 836.45 ± 49.64; PSD95 puncta: C-GFP 734.63 ± 43.90, AD-GFP 353.89 ± 24.57, AD-Nup153-GFP 500.13 ± 40.04 *P* < 0.05 ANOVA followed by SKN test, *n* = 3). Similar results were achieved in Nup153-transduced AD organoids for the sodium channel Nav1.6, which was utilized to further establish the maturation status (MFI: C-GFP 56,576 ± 3,676, AD-GFP 35,771 ± 1,701, AD-Nup153-GFP 78,713 ± 4,178, *P* < 0.05 C vs. AD, *P* < 0.001 AD vs. AD-Nup, ANOVA followed by Bonferroni test, *n* = 3).

## Discussion

In the last years, Nup153 has emerged as a platform for chromatin processes and as a key regulator of genes implicated in stemness maintenance/fate determination because of its association with chromatin co-regulators and transcription factors (e.g. Sox2, HDAC4, CTCF) [18, 22, 24]. Accordingly, in our previous work, we found that altered Nup153 levels in AD-NSCs contributed to their reduced stemness and responsiveness to differentiation stimulus. Trying to expand the protein-protein interaction (PPI) network of Nup153, which may further explain its wide action in neurogenesis and AD, we analyzed by proteomics the binding partners that co-immunoprecipitated with Nup153 in WT- and AD-NSCs. Interestingly, we found that the PPI network of Nup153 was qualitatively different between WT- and AD-NSCs indicating that the AD pathological state is characterized not only by lower protein level of Nup153 but also by its different association with binding partners. Whether this difference is due to Nup153 protein modifications or to the oxidative/proinflammatory environment of AD that affects the proteome is not known and will require further investigation.

Here we found that in WT-NSCs, Nup153 interacted with 701 proteins involved in nuclear transport as well as numerous processes that control gene expression, such as chromatin remodeling, mRNA processing, non-coding RNA, and miRNA regulation. This is in line with its upstream and extensive regulatory involvement in NSC stemness and lineage determination and its role as a hub for epigenetic processes. In addition, we observed that metabolic/biosynthetic processes were also well represented in the GO analysis of the Nup153 interacting proteins. Both the enrichment of proteins associated with gene regulation and oxidative metabolism are consistent with NSCs being metabolically active cells. Similar results were found in a proteomic study where tissue dissected from the sub-ependymal neurogenic zone was analyzed, suggesting a common proteome landscape shared by adult neural stem cells [33].

Other biological processes found to be significantly enriched from GO analysis were cell polarity, vesicle transport, actin organization, and protein targeting to the endoplasmic reticulum membrane. The endoplasmic reticulum (ER) is a network of membranes with multiple functions, such as synthesis, folding, modification, and transport of proteins. Importantly, the ER membrane is continuous with the outer membrane of the nuclear envelope and generates specific subdomains named annulate lamellae (AL), that contain nuclear pores and nucleoporins, and can be near several cytoplasmic components, increasing the interface between cytoplasmic and nucleoplasmic compartments [34, 35]. The function of AL is not completely understood. Still, evidence suggests the interaction of membrane-bound vesicles or organelles with the cytoskeleton and AL with a potential bidirectional system transport of proteins through these specialized membrane subdomains [36]. According to this preliminary evidence, Nup153 could be part of an unexplored transport mechanism through specialized membrane systems with important consequences for vesicle-based transport and the secretome.

When we analyzed the Nup153 PPI network in AD-NSCs we found that the biological processes more represented were related to mitochondrial function, energy metabolism, regulation of differentiation and protein proteasomal degradation. Mitochondrial dysfunction in AD, mainly induced by Aβ and tau production, is one of the major causes of reactive oxygen species release and losses of ATP and Ca^2+^ homeostasis with the generation of damaged oxidized proteins [37]. In physiological conditions, the ubiquitin-proteasome system controls intracellular protein quality and eliminates misfolded and damaged proteins, however its activity in AD is impaired by oxidative stress, Aβ, tau, and aggregated and cross-linked cytosolic proteins that act as proteasome inhibitors, leading to the accumulation of toxic misfolded proteins and neuronal death [38–40]. Our data suggest that alterations in energy metabolism and the ubiquitin-proteasome system characterize not only adult brain tissues [41–43] but also NSCs from the AD hippocampal niche, indicating that this landscape is a very common feature of AD involving both differentiated neurons and progenitors. This is in agreement with previous findings showing that proteasome activity is decreased in specific brain areas, such as the hippocampus, that are more susceptible to AD pathology [40].

Since our results further addressed the key role of Nup153 in the regulation of adult NSCs, we performed in vivo experiments to validate this protein as a potential therapeutic target in AD. Overexpression of Nup153 in the hippocampal niche of AD mice induced the activation, proliferation, and commitment of resident NSCs, followed by their integration in the granule cell layer of the DG. Although the transduced cells (Nup-GFP^+^) were dispersed throughout the hippocampus this was sufficient to ameliorate neurogenesis that was paralleled by improved memory. It is indeed well recognized that stem cell-based therapy’s positive effects rely not only on the direct differentiation and integration of new neurons but also on paracrine signaling by the release of soluble factors that amplify the response of resident stem cells [44]. However, we cannot exclude a positive effect of Nup153 expression on adult neurons residing in the DG. Further experiments will be necessary to explore this hypothesis.

Lately, human iPSCs derived from AD patients have been used to produce brain organoids that, thanks to their ability to organize cells similarly to the organ structure, offer new approaches to studying disease mechanisms. Both familial and sporadic AD-iPSC-derived brain organoids show AD hallmarks (Aβ and phosphorylated tau) [45], early differentiation and reduced proliferative capacity [46], synapse loss and neurodegeneration [47]. Using this approach, we observed that cortical organoids derived from sporadic AD patient’s iPSCs accumulated Aβ, nitrosative stress markers and showed an altered development with disorganized areas and fewer neuroepithelial loops than control organoids. However, following transduction of AD-iPSCs with Nup153, we obtained cerebral organoids exhibiting a better development. Specifically, AD organoids deriving from AD-iPSCs transduced with Nup153 showed the formation of neural tube-like structures and the recovery of AJ. These data indicate that Nup153 can deeply influence the developmental process and cellular organization during organogenesis in this model. This is consistent with proteomic data showing that, in murine NSCs, Nup153 interacts with multiple partners involved in the establishment of planar and apico-basal polarity, which is a mandatory prerequisite for the integrity of the neuroepithelium and cortical development [32]. Overall, these results originate from the combination of multiple approaches to model AD such as isolated mouse neural stem cells, mice and human iPSCs that have all been useful to study mechanisms and cognitive performance.

Nevertheless, looking at future translations to the clinic, is important to acknowledge that these approaches may not perfectly model the human disease. Although mouse and isolated cells are valuable in that they mimic human neurodegeneration they are mainly based on the overexpression of human proteins and may present a different susceptibility to oxidative stress and protein aggregates deriving from intrinsic species differences. On the other hand, the patient’s derived iPSCs and brain organoids offer the possibility of modelling complex cell-cell interactions in a human context. The ability of iPSCs to model AD has been debated due to the reprogramming process during their generation that may erase, at least in part, their epigenetic and age-dependent signature, a feature that is important in age-related diseases such as AD. Nonetheless, we discovered that AD iPSCs exhibited Nup153 and histone modification changes, as well as altered levels of chromatin architectural regulators such TP63 and Brd4 [48]. These findings suggest that epigenetic changes occurring in AD patients are retained even after reprogramming. Notably, increases or decreases in histone acetylation/methylation, both globally and at individual loci, characterize AD patients, experimental models and aging, and indicate a loss of heterochromatin, which is often associated with genomic instability [49].

Thus, although we are aware that these approaches may not fully model the AD pathology and that future research will be necessary to specifically address the Nup153-dependent molecular pathways, we think that Nup153 may improve cognitive performance at least by modulating neurogenesis and cellular stresses. On one hand, by modulating gene expression and interacting with many binding proteins, Nup153 function is refined and directed towards the production of new neurons that integrate in the hippocampus, the main region responsible for memory and cognition. On the other hand, Nup153 is capable of reducing the accumulation of Aβ, nitrosative stress, proteasomal degradation, and oxidative metabolism that impinge on cellular viability. Overall, this may lead to reduced neuron loss and improved cognition.

## Conclusion

Integrating biochemical and molecular studies with proteomics, we uncovered the wide role of Nup153 as an epigenetic hub in WT-NSCs and discovered that a pathological signature centered on neurodegeneration/oxidative stress characterizes the Nup153 protein network in AD-NSCs. However, what makes the Nup153 protein interaction network between WT and AD so different is not known. We can speculate that modifications of Nup153 binding affinity and thus its protein network may arise from the pro-oxidant environment of AD, but future experiments will be necessary to clarify this important point. Also, we have validated the role of Nup153 in a developmental system of human-derived 3D model of brain organoids. Although further experiments will be required to translate stem cell therapy from animal models to patients our data indicate that activation of endogenous stem cells by Nup153 modulation could be a potential approach to reduce oxidative stress and boost neurogenesis in AD.

## Electronic supplementary material

Below is the link to the electronic supplementary material.

**Supplementary Figure 1**: List of common and specific proteins identified as Nup153 interactors in WT- and AD-NSCs

**Supplementary Figure 2**: Networks of the leading **GO** Biological Processes (BP) associated with the Nup153 protein network in WT-NSCs (**A**) and AD-NSCs (**B**). Each panel reports a bipartite network consisting of two sets of nodes: one set corresponds to the leading GO BP (unlabelled nodes) found to be significantly enriched from the functional enrichment analysis (enrichment *p*-value < = 0.05) of proteins associated with Nup153 in WT- (**A**) and AD-NSCs (**B**); the other set corresponds to the proteins (labelled nodes) found to be annotated for the enriched GO BP. A protein and a GO BP term are linked if that protein is associated with/involved in that GO BP. The proteins associated to each GO BP are visualized in the network with small grey nodes. For GO BP, node size correlates with the corresponding p*-*value of the enrichment analysis (the greater the size, the greater the statistically significance) and node colors represent different categories of GO BP reported in the legend

**Supplementary Figure 3**: Network of the leading **KEGG** pathways associated with the specific Nup153 interacting proteins in AD-NSCs. This bipartite network consists of two sets of nodes: one set corresponds to the leading KEGG pathways (unlabelled nodes) found to be significantly enriched from the functional enrichment analysis (enrichment *p*-value < = 0.05) and the other set corresponds to the proteins (labelled nodes) that were found to be annotated for the enriched KEGG pathways. A protein and a KEGG pathways are linked if that protein is associated with/involved in that KEGG pathways. The proteins associated with each KEGG pathway are visualized in the network with small grey nodes. For pathways, node size correlates with the corresponding p*-*value of the enrichment analysis (the greater the size, the greater the statistically significance) and node colors represent different categories of KEGG pathways reported in the legend

**Supplementary Figure 4**: (**A**) Confocal images showing iPSCs from healthy controls and AD patients labelled for the stem cell markers Sox2, TRA-1, Oct4, Nanog and SSEA4. (**B**) Western blot evaluation of Nanog, Oct4 and Sox2 in control and AD iPSCs and relative quantification. Differences were found in Sox2 levels. (**C-D**) Confocal analysis and western blot showing the levels of Nup153 in iPSCs from control and AD samples. *P < 0.05, ** *P* < 0.01

**Supplementary Figure 5**: (**A-B**) Western blot evaluation of chromatin regulators and histone modifications in control and AD iPSCs with the relative quantification. Optical density values of western blot data were calculated on the base of actin expression. (**C**) Representative images of Brd4 expression in control and AD iPSC at lower (scale bar 10 μm) and higher magnification (scale bar 5 μm; projections X-Y and Y-Z) and relative quantification of the mean fluorescence intensity (MFI). (**D**) Confocal analysis showing the level of Nup153 in iPSCs from control and AD samples with or without Nup153 transduction (*n* = 3, Scale bar 50 μm). Nuclei were counterstained with DAPI. E) Bright field images of embryoid bodies derived from control and AD-iPSCs transduced with GFP or Nup153-GFP (scale bar 100 μm). *P < 0.05, ** *P* < 0.01, *** *P* < 0.01

**Supplementary Figure 6**: (**A-B**) Nitro-tyrosine (N-Tyr) and cysteine-S-nitrosylation (SNO-cys) levels evaluated by dot blot analysis (*n* = 3) in control, AD and AD-Nup organoids. Each lysate was obtained from the pool of 3–4 individual organoids. Hippocampal lysate from 9-month-old 3×Tg mice was used as positive control. Red ponceau (RP) staining was used as loading index and used to normalize samples. (**C**) Immunolabelling with Nav1.6 and Sox2 antibodies in control, AD and AD-Nup organoids and relative quantification of the mean fluorescence intensity (MFI) of the sodium channel Nav1.6. Nuclei were counterstained with DAPI. Scale bar 20 μm, *P < 0.05, ***P < 0.001

**Supplemental Fig. 7**: List of full-length blots
**Supplementary Table 1**: List of selected GO processes, the corresponding annotated proteins and relative *p* values in WT-NSCs, AD-NSCs and AD-Nup-NSCs

**Supplementary Material 8**: Data relative to supplementary figures

## Data Availability

All data generated or analysed during this study are included in this published article and in its supplementary information files.

## Abbreviations

3×Tg: Triple transgenic model of AD
AD: Alzheimer’s disease
AD-NSCs: AD-neural stem cells
Aβ: Amyloid-β
AJ: Adherent junctions
BrdU: Bromodeoxyuridine
DCX: Doublecortin
DG: Dentate gyrus
DHE: Dihydroethidium
ER: Endoplasmic reticulum
GFP: Green fluorescent protein
GO: Gene ontology
HATs: Histone acetyl transferases
HDACs: Histone deacetylases
iPSCs: Inducible pluripotent stem cells
KEGG: Kyoto Encyclopedia of Genes and Genomes
MFI: mean fluorescence intensity
MWM: Morris Water Maze
NeuN: Neuronal N
NSC: Neural stem cell
Nup153: Nucleoporin153
OCT: Optimal cutting temperature compound
P-NCAM: Polysialic Acid-NCAM
PPI: Protein-protein interaction
SGZ: Subgranular zone
SVZ: Subventricular zone
