# Supplementary figures and images for: Nucleoporin 153 deficiency in adult neural stem cells defines a pathological protein-network signature and defective neurogenesis in a mouse model of AD
Source: Stem Cell Res Ther. 2024 Sep 3;15:275. doi: 10.1186/s13287-024-03805-1 (PMC11373261; doi:10.1186/s13287-024-03805-1)

**A**

## WT-specific GO BP

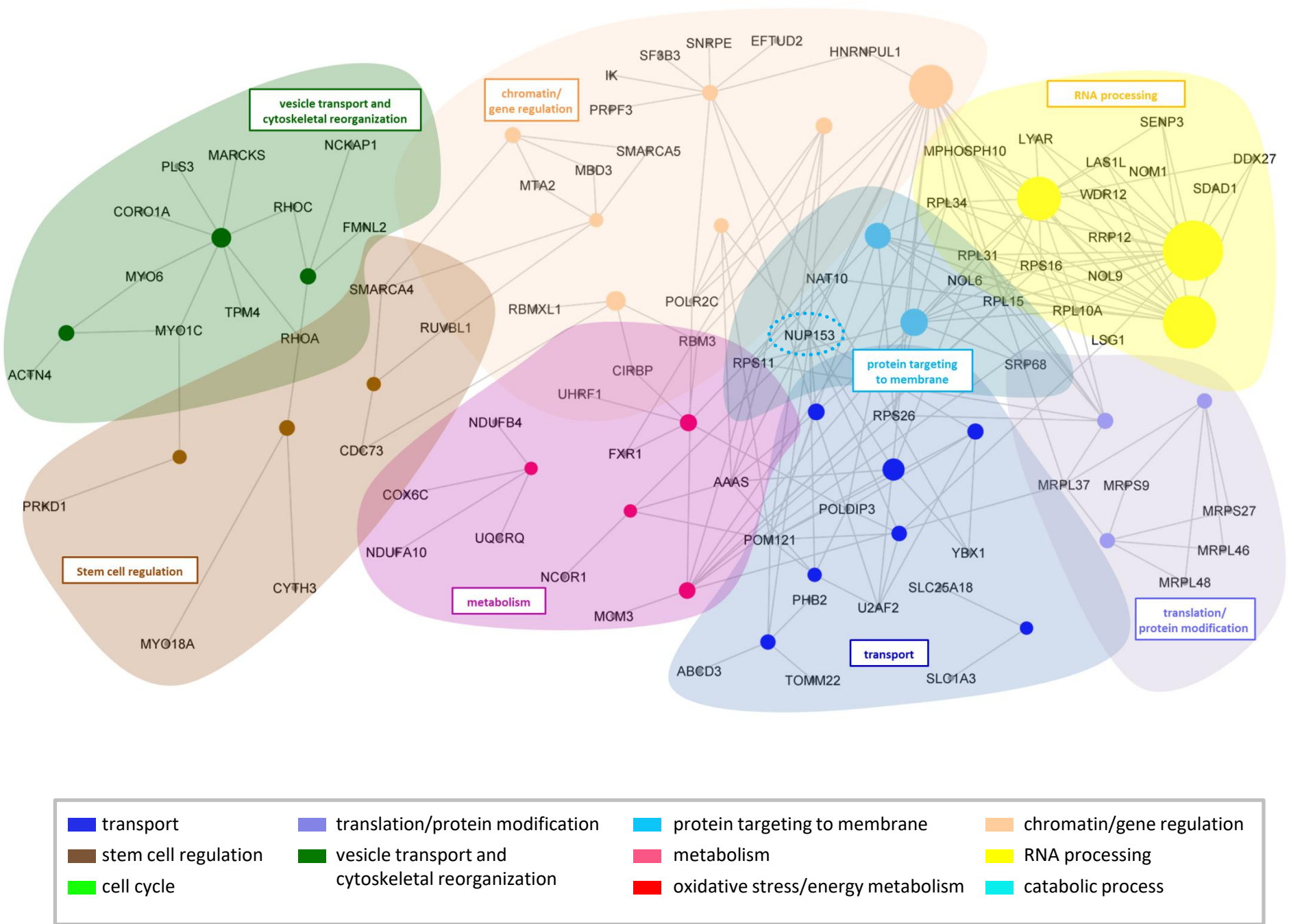**B**

## AD-specific GO BP

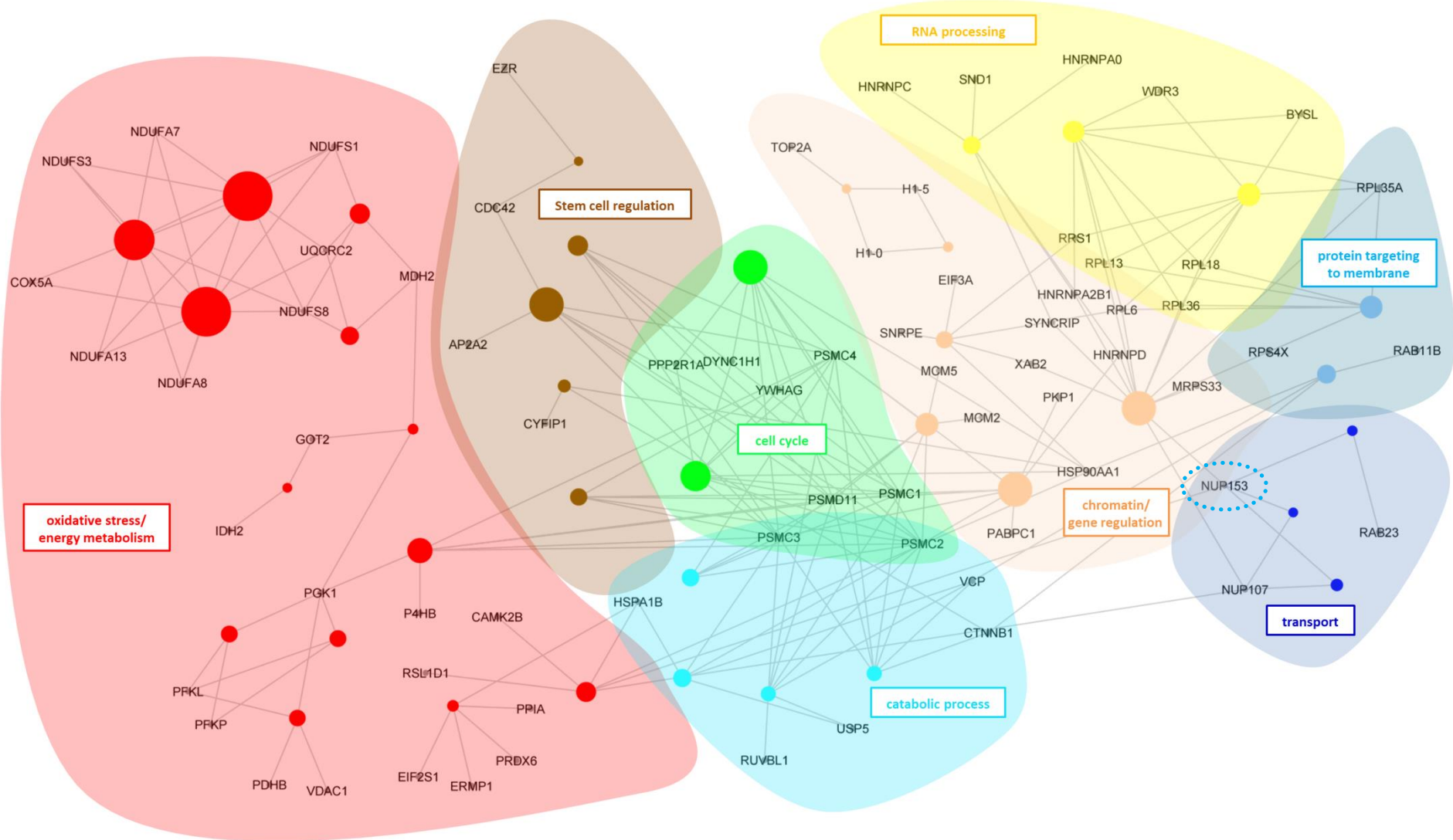

Supplement: Supplementary file 2 — Supplementary Figure 2: Networks of the leading GO Biological Processes (BP) associated with the Nup153 protein network in WT-NSCs (A) and AD-NSCs (B). Each panel reports a bipartite network consisting of two sets of nodes: one set corresponds to the leading GO BP (unlabelled nodes) found to be significantly enriched from the functional enrichment analysis (enrichment p-value < = 0.05) of proteins associated with Nup153 in WT- (A) and AD-NSCs (B); the other set corresponds to the proteins (labelled nodes) found to be annotated for the enriched GO BP. A protein and a GO BP term are linked if that protein is associated with/involved in that GO BP. The proteins associated to each GO BP are visualized in the network with small grey nodes. For GO BP, node size correlates with the corresponding p-value of the enrichment analysis (the greater the size, the greater the statistically significance) and node colors represent different categories of GO BP reported in the legend [file 13287_2024_3805_MOESM2_ESM.pdf]

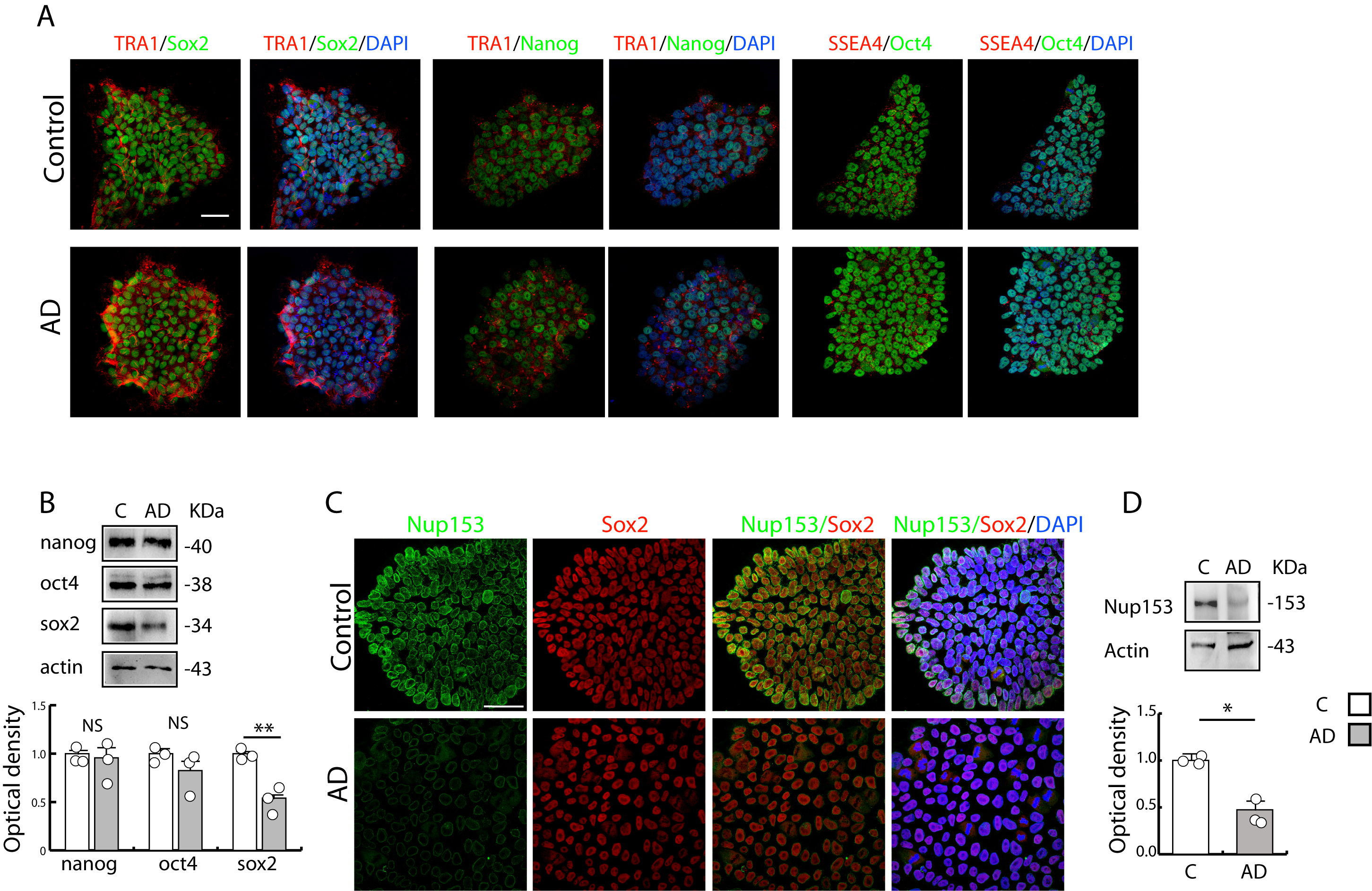

Supplement: Supplementary file 4 — Supplementary Figure 4: (A) Confocal images showing iPSCs from healthy controls and AD patients labelled for the stem cell markers Sox2, TRA-1, Oct4, Nanog and SSEA4. (B) Western blot evaluation of Nanog, Oct4 and Sox2 in control and AD iPSCs and relative quantification. Differences were found in Sox2 levels. (C-D) Confocal analysis and western blot showing the levels of Nup153 in iPSCs from control and AD samples. *P < 0.05, ** P < 0.01 [file 13287_2024_3805_MOESM4_ESM.png]

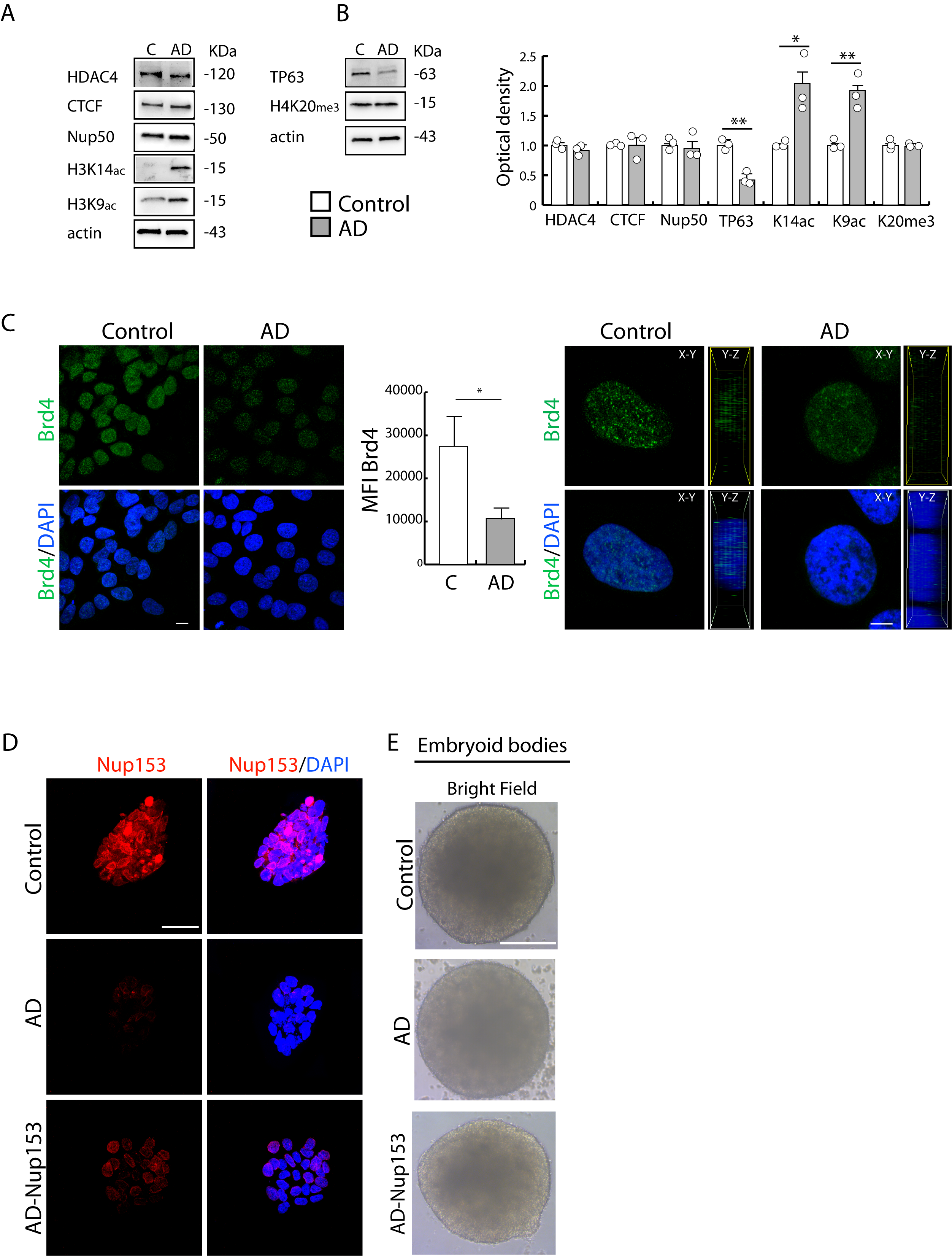

Supplement: Supplementary file 5 — Supplementary Figure 5: (A-B) Western blot evaluation of chromatin regulators and histone modifications in control and AD iPSCs with the relative quantification. Optical density values of western blot data were calculated on the base of actin expression. (C) Representative images of Brd4 expression in control and AD iPSC at lower (scale bar 10 μm) and higher magnification (scale bar 5 μm; projections X-Y and Y-Z) and relative quantification of the mean fluorescence intensity (MFI). (D) Confocal analysis showing the level of Nup153 in iPSCs from control and AD samples with or without Nup153 transduction (n = 3, Scale bar 50 μm). Nuclei were counterstained with DAPI. E) Bright field images of embryoid bodies derived from control and AD-iPSCs transduced with GFP or Nup153-GFP (scale bar 100 μm). *P < 0.05, ** P < 0.01, *** P < 0.01 [file 13287_2024_3805_MOESM5_ESM.png]

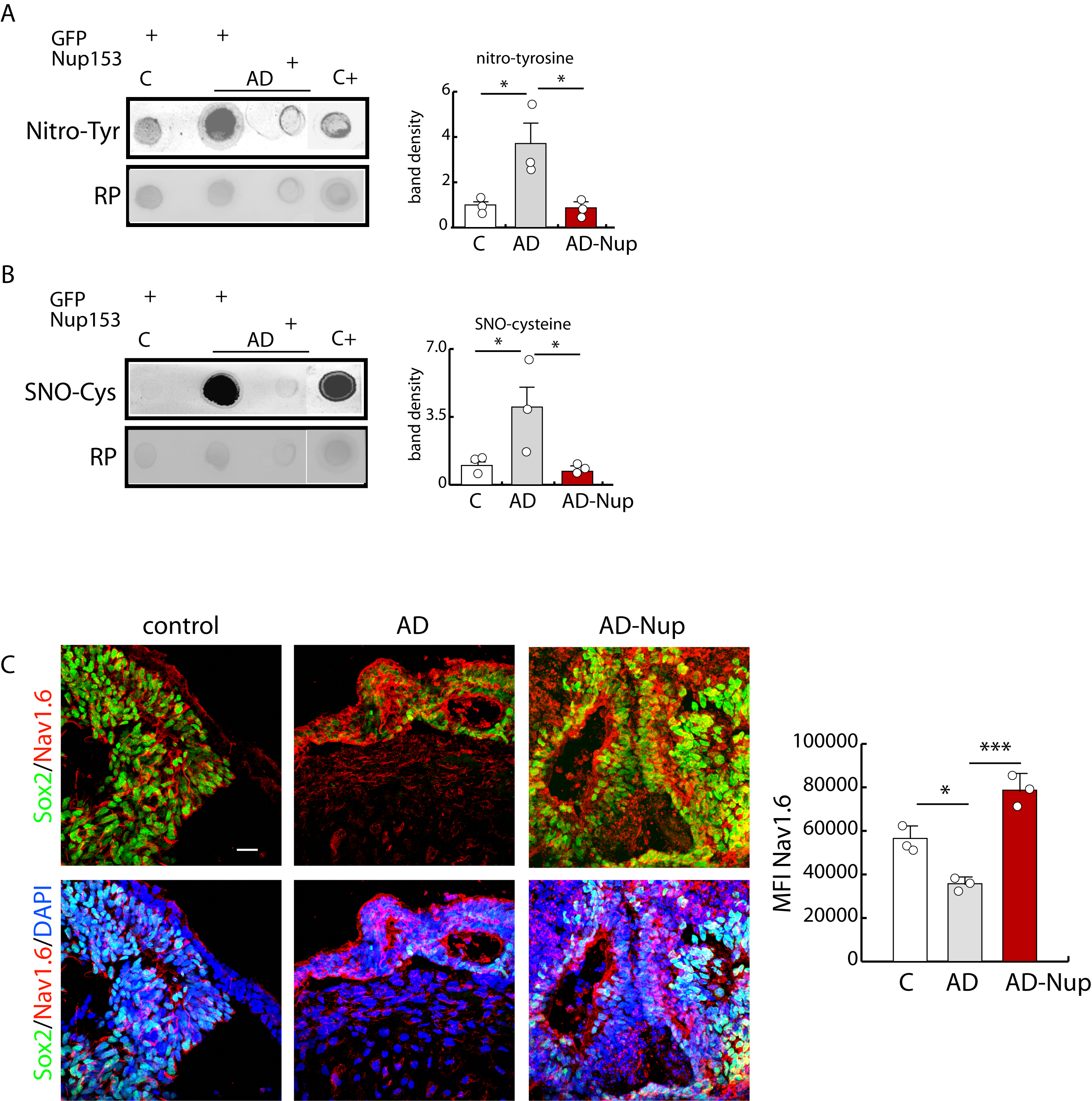

Supplement: Supplementary file 6 — Supplementary Figure 6: (A-B) Nitro-tyrosine (N-Tyr) and cysteine-S-nitrosylation (SNO-cys) levels evaluated by dot blot analysis (n = 3) in control, AD and AD-Nup organoids. Each lysate was obtained from the pool of 3–4 individual organoids. Hippocampal lysate from 9-month-old 3×Tg mice was used as positive control. Red ponceau (RP) staining was used as loading index and used to normalize samples. (C) Immunolabelling with Nav1.6 and Sox2 antibodies in control, AD and AD-Nup organoids and relative quantification of the mean fluorescence intensity (MFI) of the sodium channel Nav1.6. Nuclei were counterstained with DAPI. Scale bar 20 μm, *P < 0.05, ***P < 0.001 [file 13287_2024_3805_MOESM6_ESM.png]
