# Supplementary material for: Nucleoporin 153 deficiency in adult neural stem cells defines a pathological protein-network signature and defective neurogenesis in a mouse model of AD
Source: Stem Cell Res Ther. 2024 Sep 3;15:275. doi: 10.1186/s13287-024-03805-1 (PMC11373261; doi:10.1186/s13287-024-03805-1)

AD-specific KEGG

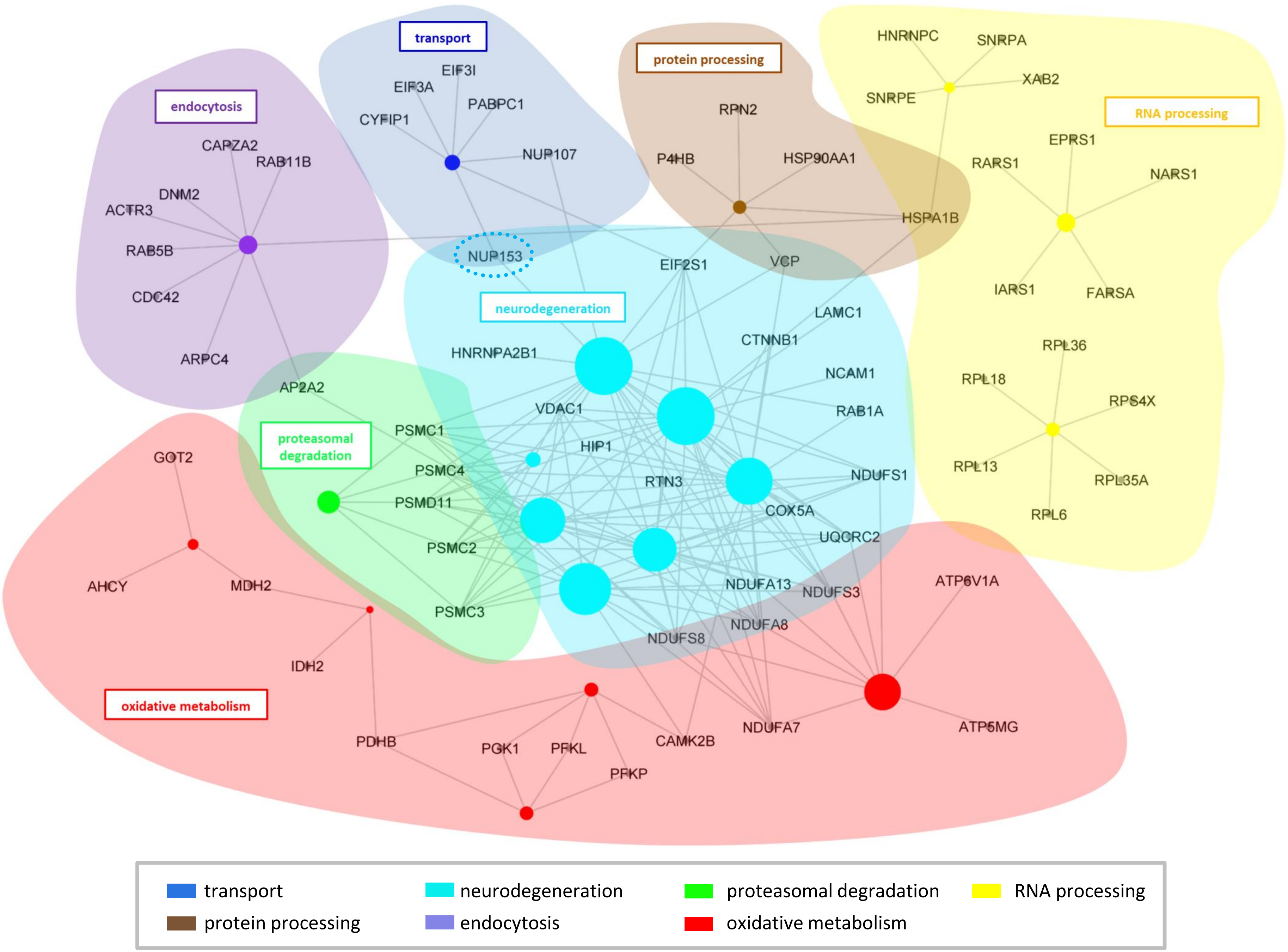

Supplement: Supplementary file 3 — Supplementary Figure 3: Network of the leading KEGG pathways associated with the specific Nup153 interacting proteins in AD-NSCs. This bipartite network consists of two sets of nodes: one set corresponds to the leading KEGG pathways (unlabelled nodes) found to be significantly enriched from the functional enrichment analysis (enrichment p-value < = 0.05) and the other set corresponds to the proteins (labelled nodes) that were found to be annotated for the enriched KEGG pathways. A protein and a KEGG pathways are linked if that protein is associated with/involved in that KEGG pathways. The proteins associated with each KEGG pathway are visualized in the network with small grey nodes. For pathways, node size correlates with the corresponding p-value of the enrichment analysis (the greater the size, the greater the statistically significance) and node colors represent different categories of KEGG pathways reported in the legend [file 13287_2024_3805_MOESM3_ESM.pdf]
