# Supplementary material for: Nucleoporin 153 deficiency in adult neural stem cells defines a pathological protein-network signature and defective neurogenesis in a mouse model of AD
Source: Stem Cell Res Ther. 2024 Sep 3;15:275. doi: 10.1186/s13287-024-03805-1 (PMC11373261; doi:10.1186/s13287-024-03805-1)

Data relative to figure 1: Gel showing the pattern of samples co-immunoprecipitated with Nup153 used in proteomic analysis

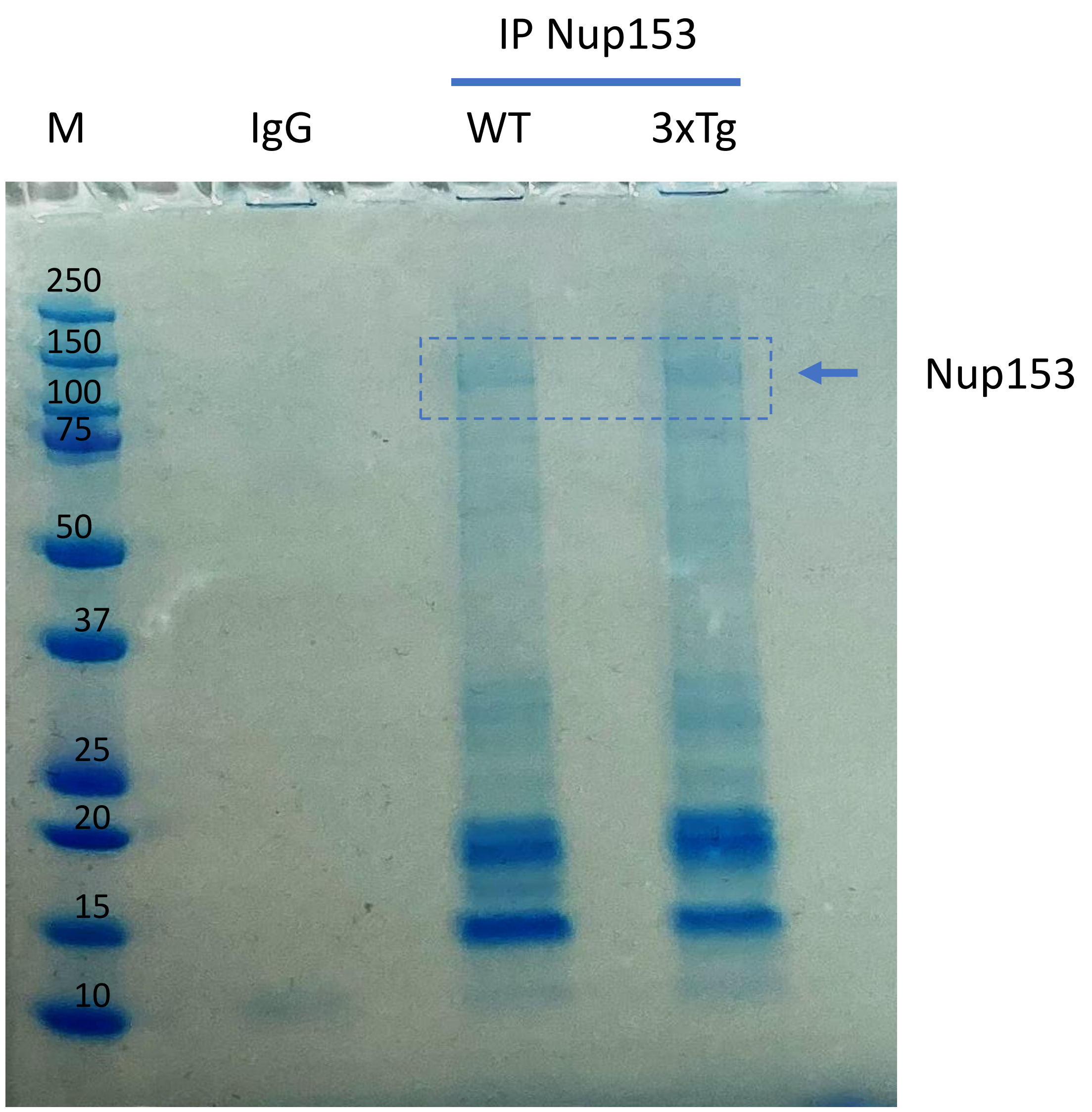

Data relative to figure 5

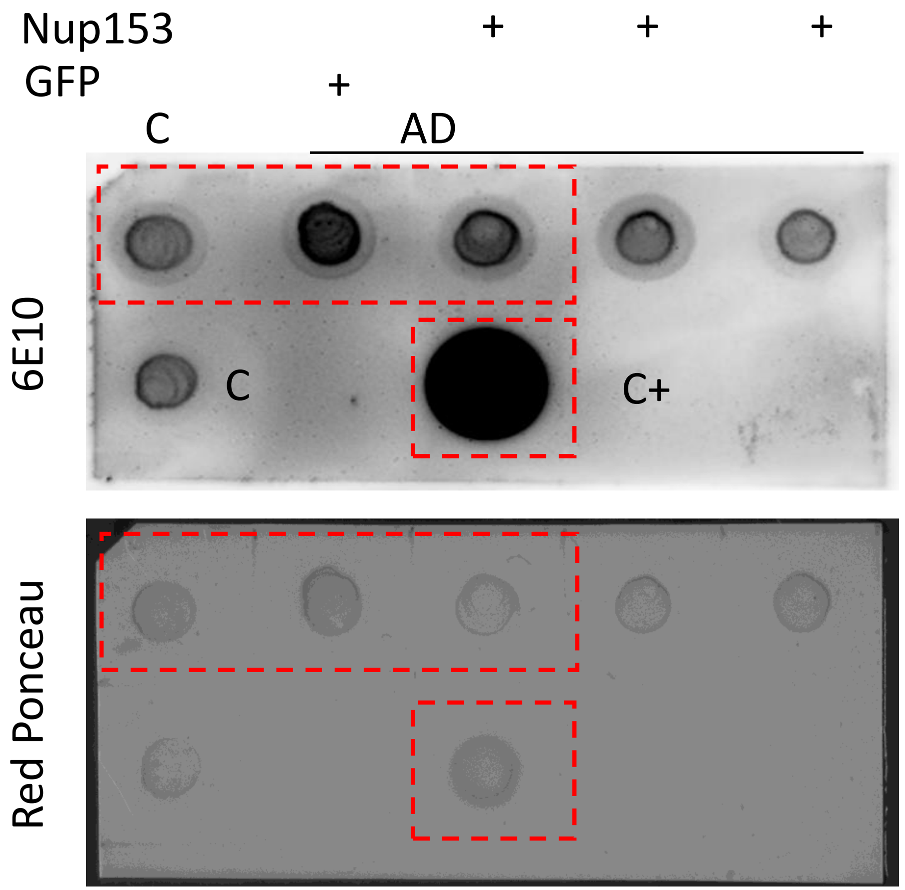

Data relative to figure 6

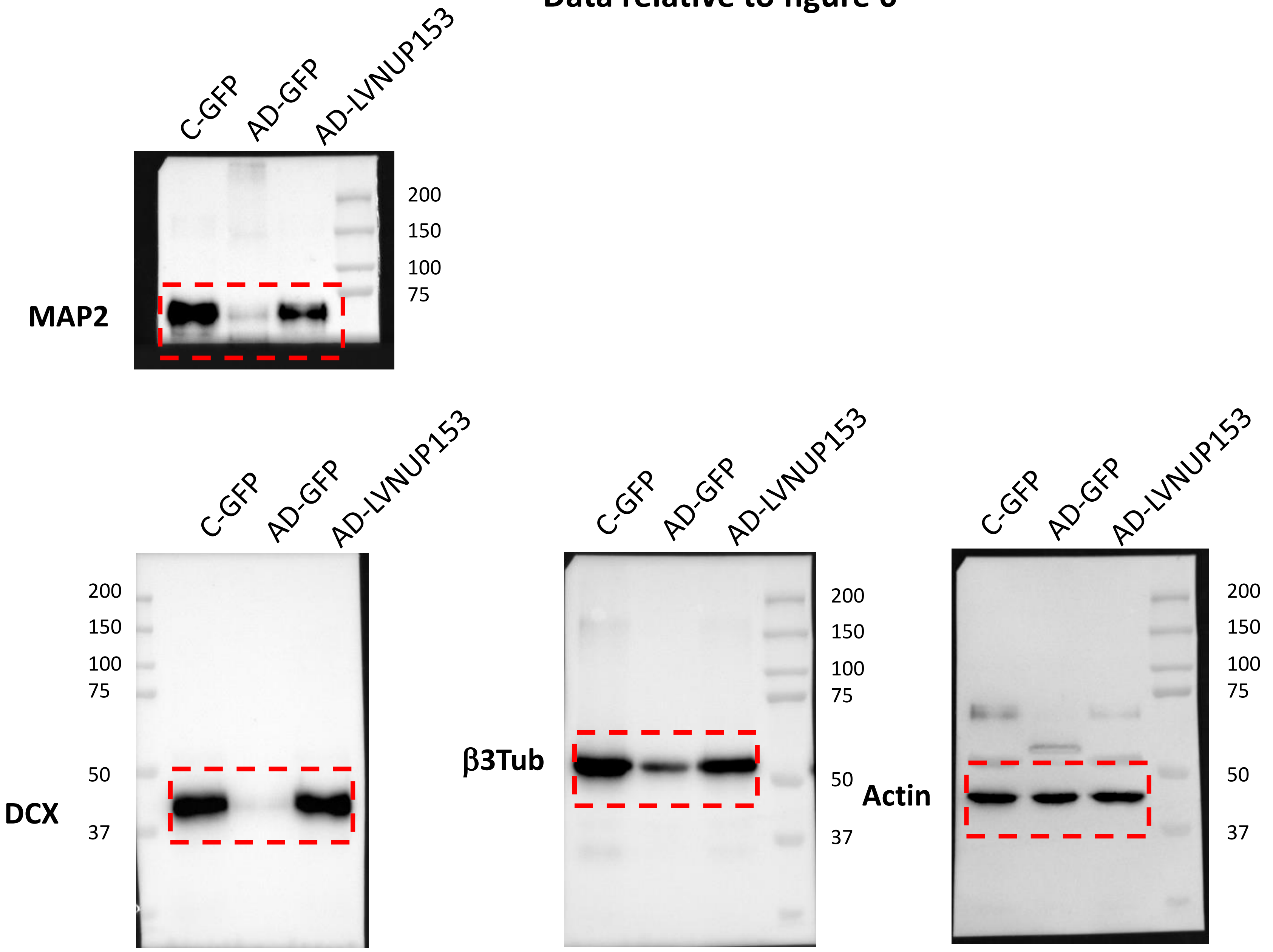

Data relative to figure 6

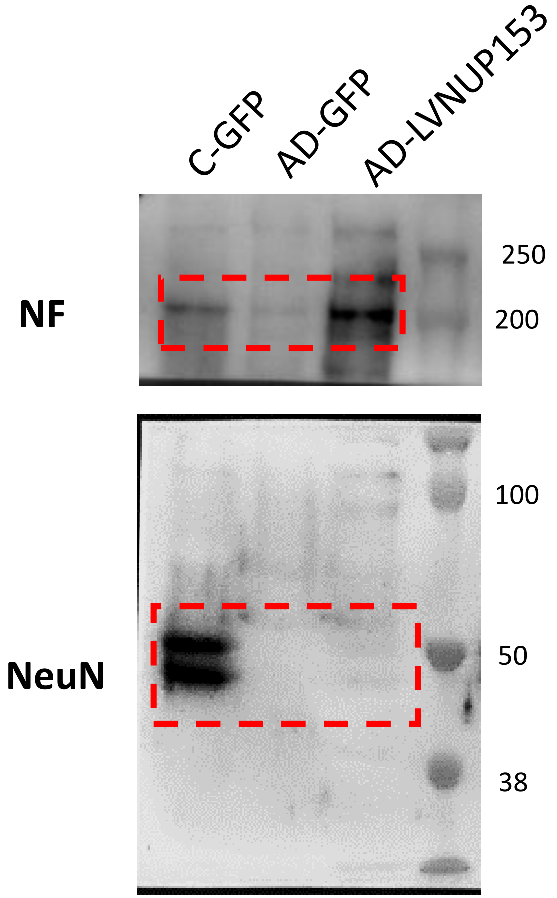

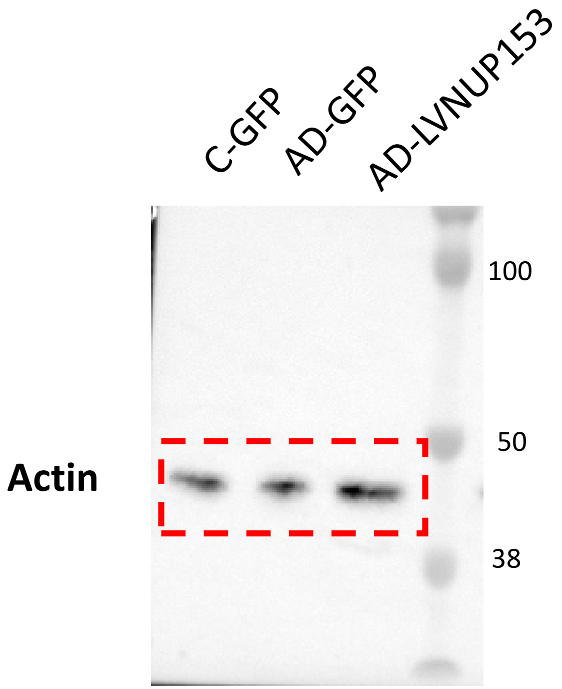

Data relative to Supplemental figure 4

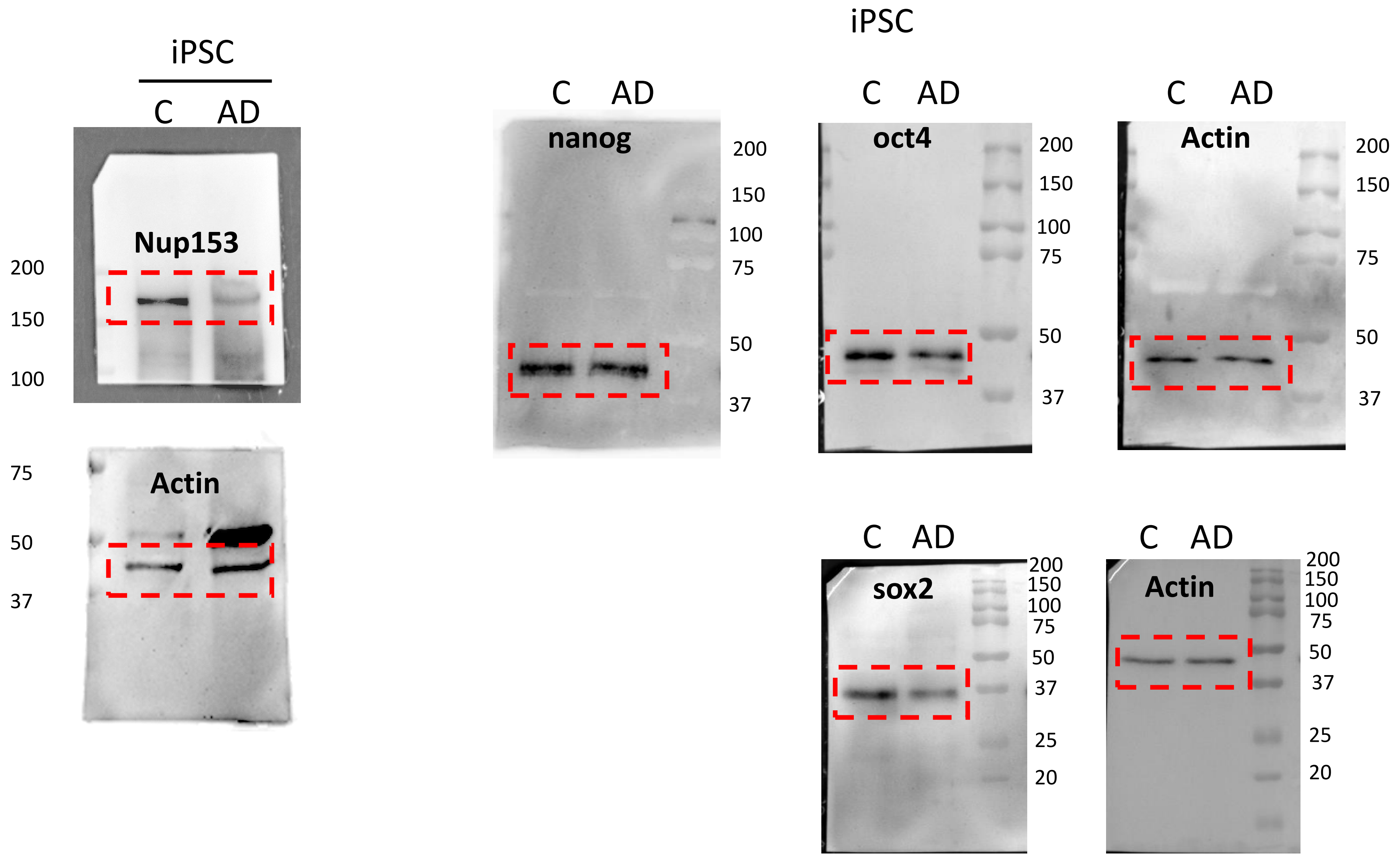

Data relative to Supplemental figure 5

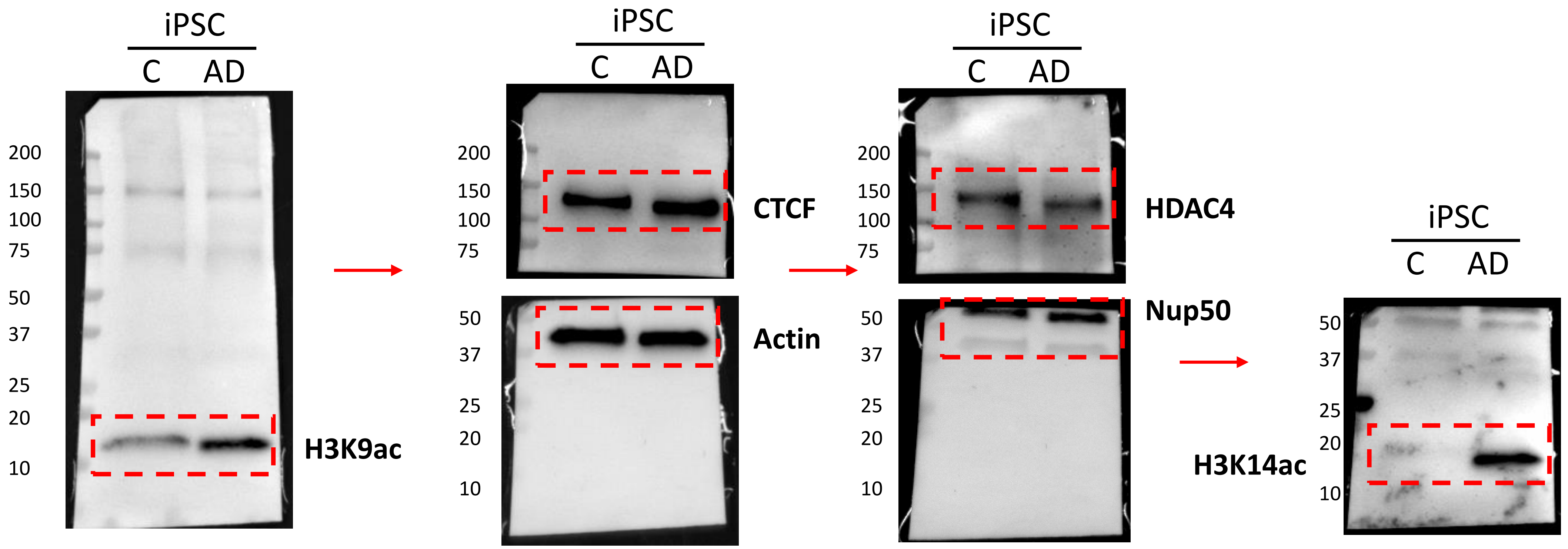

Data relative to Supplemental figure 5

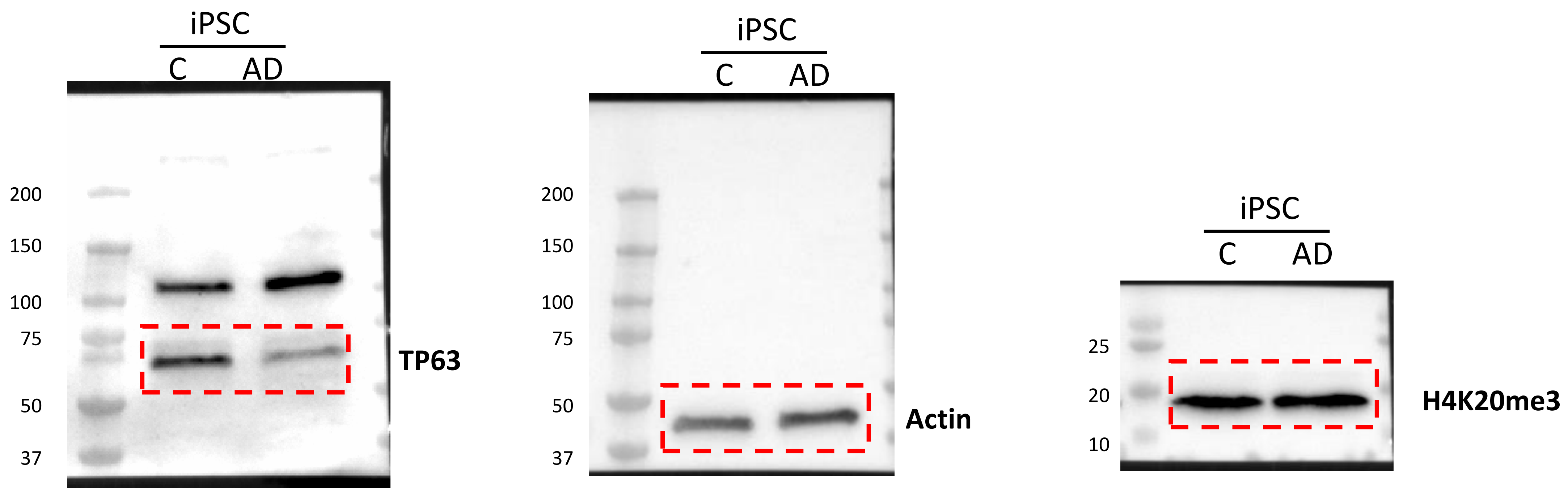

Data relative to Supplemental figure 6

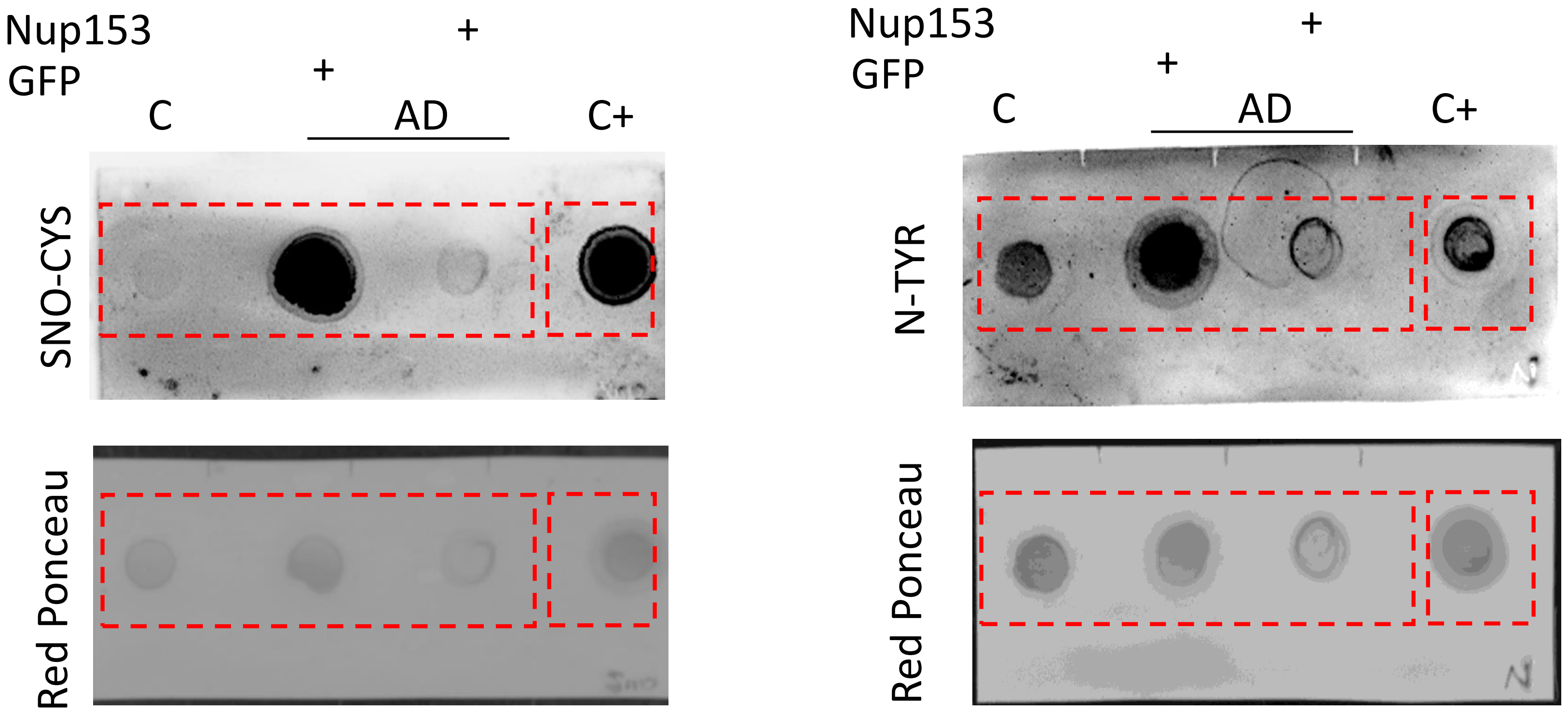

Supplement: Supplementary file 8 — Supplementary Material 8: Data relative to supplementary figures [file 13287_2024_3805_MOESM8_ESM.pdf]
